# Cytokine pattern in patients with ST-elevation myocardial infarction treated with the interleukin-6 receptor antagonist tocilizumab

**DOI:** 10.1136/openhrt-2023-002301

**Published:** 2023-08-17

**Authors:** Sindre Woxholt, T Ueland, Pål Aukrust, Anne Kristine Anstensrud, Kaspar Broch, Ingvild Maria Tøllefsen, Liv Ryan, Bjørn Bendz, Einar Hopp, Nils-Einar Kløw, Ingebjørg Seljeflot, Bente Halvorsen, Tuva B Dahl, Camilla Huse, Geir Øystein Andersen, Lars Gullestad, Rune Wiseth, Brage H Amundsen, Jan Kristian Damas, Ola Kleveland

**Affiliations:** 1 Clinic of Cardiology, St Olavs Hospital Trondheim University Hospital, Trondheim, Norway; 2 Department of Circulation and Medical Imaging, Norwegian University of Science and Technology, Trondheim, Norway; 3 Research Institute of Internal Medicine, Rikshospitalet University Hospital, Oslo, Norway; 4 K. G. Jebsen Thrombosis Research and Expertise Center (TREC), UiT The Arctic University of Norway, Tromso, Norway; 5 Faculty of Medicine, Institute of Clinical Medicine, University of Oslo, Oslo, Norway; 6 Section of Clinical Immunology and Infectious Disease, Oslo Universitetssykehus, Oslo, Norway; 7 Research Institute of Internal Medicine, Rikshospitalet Research Institute for Internal Medicine, Oslo, Norway; 8 Department of Cardiology, Oslo University Hospital, Oslo, Norway; 9 Department of Cardiology, Rikshospitalet University Hospital, Oslo, Norway; 10 K. G. Jebsen Cardiac Research Centre and Centre for Heart Failure Research, University of Oslo, Oslo, Norway; 11 Department of clinical and Molecular medicine, Norwegian University of Science and Technology, Trondheim, Norway; 12 Department of Radiology and Nuclear Medicine, Rikshospitalet University Hospital, Oslo, Norway; 13 Department of Radiology, Oslo University Hospital Ullevaal, Oslo, Norway; 14 Center for Clinical Heart Research, Department of Cardiology, Oslo University Hospital Ullevaal, Oslo, Norway; 15 Department of Cardiology, Oslo universitetssykehus Ulleval, Oslo, Norway; 16 Department of Cardiology, Center for Clinical Heart Research, University of Oslo, Oslo, Norway; 17 Department of Infectious Diseases, St Olavs Hospital Trondheim University Hospital, Trondheim, Norway; 18 Department of Clinical and Molecular Medicine, Centre of Molecular Inflammation Research, Norwegian University of Science and Technology, Trondheim, Norway

**Keywords:** Inflammation, Myocardial Infarction, STEMI, Biomarkers

## Abstract

**Background:**

Tocilizumab improves myocardial salvage index (MSI) in patients with ST-elevation myocardial infarction (STEMI), but its mechanisms of action are unclear. Here, we explored how cytokines were affected by tocilizumab and their correlations with neutrophils, C-reactive protein (CRP), troponin T, MSI and infarct size.

**Methods:**

STEMI patients were randomised to receive a single dose of 280 mg tocilizumab (n=101) or placebo (n=98) before percutaneous coronary intervention. Blood samples were collected before infusion of tocilizumab or placebo at baseline, during follow-up at 24–36, 72–168 hours, 3 and 6 months. 27 cytokines were analysed using a multiplex cytokine assay. Cardiac MRI was performed during hospitalisation and 6 months.

**Results:**

Repeated measures analysis of variance showed significant (p<0.001) between-group difference in changes for IL-6, IL-8 and IL-1ra due to an increase in the tocilizumab group during hospitalisation. IL-6 and IL-8 correlated to neutrophils in the placebo group (r=0.73, 0.68, respectively), which was attenuated in the tocilizumab group (r=0.28, 0.27, respectively). A similar pattern was seen for MSI and IL-6 and IL-8 in the placebo group (r=−0.29, –0.25, respectively) in patients presenting ≤3 hours from symptom onset, which was attenuated in the tocilizumab group (r=−0.09,–0.14, respectively).

**Conclusions:**

Tocilizumab increases IL-6, IL-8 and IL-1ra in STEMI. IL-6 and IL-8 show correlations to neutrophils/CRP and markers of cardiac injury in the placebo group that was attenuated in the tocilizumab group. This may suggest a beneficial effect of tocilizumab on the ischaemia-reperfusion injury in STEMI patients.

**Trial registration number:**

NCT03004703.

**WHAT IS ALREADY KNOWN ON THIS TOPIC**

- Inflammation plays an important role in the development of atherosclerosis, myocardial infarction and ischaemia-reperfusion injury. IL-1β inhibition reduces future cardiovascular events in patients with previous MI (Canakinumab Anti-Inflammatory Thrombosis Outcome Study), while IL-6 inhibition improves myocardial salvage index in ST-elevation myocardial infarction (STEMI) patients (ASSessing the effect of Anti-IL-6 treatment in Myocardial Infarction study), but the underlying mechanisms are uncertain.

**WHAT THIS STUDY ADDS**

- This study shows that tocilizumab leads to an increase in IL-6, IL-8 and IL-1ra in patients with STEMI, and it attenuates the correlation of IL-6 and IL-8 with neutrophils/C reactive protein and markers of myocardial injury observed in the placebo group.

**HOW THIS STUDY MIGHT AFFECT RESEARCH, PRACTICE OR POLICY**

- The findings suggest a potential beneficial effect of tocilizumab on ischaemia-reperfusion injury in STEMI patients and may provide valuable data for further research efforts aimed at improving clinical outcomes and guiding the development of targeted therapies for STEMI patients.

## Introduction

In patients with myocardial infarction (MI), inflammation plays a pivotal role in various detrimental processes, including endothelial dysfunction, plaque instability, thrombosis and impaired tissue healing. It is a key factor in the development of atherosclerosis, the underlying cause of MI. Moreover, inflammation has been closely associated with critical outcomes such as infarct size, left ventricular remodelling, the development of heart failure and overall mortality.^1 2^ Whereas an inflammatory response is essential to clear necrotic debris and promote healing and scar tissue in MI, prolonged or exaggerated inflammation could have detrimental effects.

The interleukin (IL)−1 → IL-6 → C reactive protein (CRP) axis has been suggested to play an important role in cardiovascular inflammation.^3^ Elevated CRP predicts future coronary events and is associated with worse outcomes in acute coronary syndromes.^4 5^ Moreover, the landmark Canakinumab Anti-Inflammatory Thrombosis Outcome Study showed that IL-1β inhibition reduces the risk of future cardiovascular events in patients with previous MI and residual inflammatory risk defined as CRP>2 mg/L. This reduction was most prominent in patients where canakinumab reduced IL-6 levels.^6^

We have previously shown that a single dose of the IL-6 receptor antagonist tocilizumab significantly reduced CRP levels and mitigated troponin T (TnT) release in patients with non-ST segment elevation MI (NSTEMI).^7^ These effects were most prominent in patients treated with percutaneous coronary intervention (PCI), suggesting a beneficial effect on periprocedural myocardial injury. In the ASSessing the effect of Anti-IL-6 treatment in Myocardial Infarction (ASSAIL-MI) trial, we showed that tocilizumab increased the myocardial salvage index (MSI) evaluated by cardiac MR imaging (CMR) in patients with ST-segment elevation MI (STEMI). This effect was accompanied by a reduction in CRP.^8^ These results suggest that tocilizumab may have a cardioprotective effect in MI by mitigating the ischaemia-reperfusion injury.

We have previously shown that tocilizumab increases the CC chemokine macrophage inflammatory protein (MIP-1β/CCL4) and the CXC chemokine interferon-γ inducible protein (IP-10/CXCL10) in NSTEMI patients.^9^ The inflammatory pattern may, however, be different in STEMI and NSTEMI.^10 11^ We have recently shown that tocilizumab reduces neutrophil counts and function in STEMI, but data on cytokine levels in plasma/serum are lacking.^12^ In this study, our aim was to investigate the impact of tocilizumab on a comprehensive range of pro and anti-inflammatory cytokines in patients with STEMI. Additionally, we sought to explore the associations between these cytokines, myocardial damage measured by TnT and CMR, aiming to shed light on the underlying mechanisms of ischaemia-reperfusion injury and how these were modulated by tocilizumab.

## Methods

### Study population and design

This was a predefined substudy of the phase II, parallel arm, double-blind, randomised placebo-controlled, multicentre ASSAIL-MI trial (ClinicialTrials.gov, NCT03004703). The trial was performed at Oslo University Hospital Rikshospitalet, Oslo University Hospital Ullevål and St. Olavs Hospital, Trondheim, Norway.

Eligible patients (between 18 and 80 years) were allocated in a 1:1 fashion to receive a single 1-hour intravenous infusion of 280 mg of tocilizumab or placebo. The infusion was started as soon as possible after obtaining oral consent from the patients, and the mean time from the start of the infusion to wire crossing was 8 min. The administration of tocilizumab/placebo did not delay the opening of the infarct related artery. This dose has been shown to achieve complete IL-6 receptor inhibition for 2–3 weeks.^13^ Additionally, the same dose led to significant suppression of CRP in patients with NSTEMI who were treated with 280 mg tocilizumab.^7^ Treatment allocation was stratified by time from symptom onset (≤3 or >3 hours). The inclusion criteria were STEMI with symptom onset less than 6 hours before PCI. Exclusion criteria were previous MI, cardiogenic shock, resuscitated cardiac arrest, left bundle branch block, renal or liver failure, current or chronic infection, current or chronic autoimmune/ inflammatory disease, recent major surgery or immunosuppressive treatment other than low-dose corticosteroids ≤prednisone 5 mg/daily.

The primary endpoint of the ASSAIL-MI trial was MSI ($\frac{area\;\;at\;\;risk-infarct\;\;size}{area\;\;at\;\;risk}\times 100$) assessed by CMR. The complete trial protocol is described elsewhere.^8^

### Blood sampling protocol

Baseline blood samples were collected from the arterial sheath prior to infusion of the study drug/placebo. Peripheral venous blood samples were collected at 24–36 hours and 72–168 hours and at 3 and 6 months. The blood was drawn into tubes containing ethylenediaminetetraacetic acid that were immediately centrifuged at 2000 g relative centrifugal force for 20 min at 4°C. The plasma was separated into multiple aliquots (~1 mL) and stored at −80°C. The samples were thawed only once.

### Cytokine measurements

The plasma was analysed for 27-cytokines (Bio-Plex Pro Human Cytokine 27-plex Assay) in single replicate using Luminex xMap Technology on a Bio-Plex 200 system (Bio-Rad Laboratories, California, USA). This assay quantifies IL-1β, IL-1 receptor antagonist (IL-1ra), IL-2, IL-4, IL-5, IL-6, IL-7, IL-8/CXCL-8, IL-9, IL-10, IL-12 (p70), IL-13, IL-15, IL-17, basic fibroblast growth factor (b-FGF), granulocyte colony stimulating factor (G-CSF), granulocyte-macrophage colony-stimulating factor (GM-CSF), interferon-gamma (IFN-ɣ), eotaxin/CCL11, IP-10/CXCL10, macrophage chemoattractant protein/CCL2, MIP-1α/CCL3, MIP-1β/CCL4, (RANTES)/CCL5, tumour necrosis factor (TNF), platelet-derived growth factor (PDGF) and vascular endothelial growth factor.

The assay uses detection antibodies for the biomarker of interest, conjugated with a fluorescent indicator. The acquired fluorescent intensity is used to calculate the concentration (pg/mL) of each cytokine using a standard curve. The standard dilution series curves for b-FGF, IL-10, IL-15 and IL-17 were distorted, and the results were not credible. These results were therefore discarded, and not included in the analyses. Interassay coefficient of variations (CV) was <15% for all analytes (mean 5.13%), and most intra-assay CVs were <11% (mean 5.10%). Many of our samples were out of range (OOR) in respect to the manufacturer’s tolerance. To obtain continuous datasets, we established a new global standard curve that was used to calculate the concentration of all cytokines with >10% of the values OOR. For cytokines with <10% of values OOR, we replaced the missing values using the lowest measured concentration for that cytokine.

### Routine laboratory analyses

Leucocytes and differential counts were analysed per routine. High sensitivity CRP was analysed by an immunoturbidimetric method using the MODULAR platform (Roche Diagnostics, Basel, Switzerland) and Siemens Advia Chemistry XPT (Siemens Healthcare, Erlangen, Germany). High sensitivity TnT was analysed by electrochemiluminescence immunoassay using Roche Elecsys 2010 and Roche Cobas 8000 (Roche Diagnostics, Basel, Switzerland).

### Cardiac MR

CMR was performed at 3–7 days and 6 months after MI. MSI was calculated as the difference between early enhancement volume and late enhancement volume on short axis slices 3–7 days after MI. MI volume was calculated as the sum of late enhancement volume on short axis slices at 3–7 days and 6 months. Images were analysed by using Segment.^14^

### Statistics

The primary aim was to assess group differences in cytokine levels between tocilizumab and placebo, in patients with STEMI treated by primary PCI. A mixed between-within subjects analysis of variance was performed to assess the impact of the intervention (between group—tocilizumab vs placebo) and the time interaction (within groups—multiple timepoints). Due to multiple comparisons, we applied Bonferroni correction (α=0.05/23 cytokines) resulting in a cut-off p value of 0.002 for postanalysis.

The cytokines with a group×time interaction p<0.002 were analysed for between-group differences in changes from baseline using an independent sample t-test. Pearson correlation coefficient was calculated for the area under the curve (AUC) for the same cytokines against MSI (the primary endpoint in the main study) and infarct size, TnT, CRP (all secondary aims in the main study) and neutrophils. For both the temporal assessment of cytokines and for correlations with endpoints, we also performed sensitivity analysis according to symptom duration (≤3 or > 3 hours) as stratified in the main study. Apart from the initial between-within subjects analysis of variance, a two-sided p<0.05 was considered significant.

Interleukin-9 and MIP-1β were normally distributed, otherwise all results were log-transformed to reach near normal distribution.

IBM SPSS Statistics V.28.0.1.0 (142) was used for the analysis.

## Results

### Baseline characteristics

Two hundred patients were included between 16 March 2017 and 13 February 2020. One patient withdrew consent. Accordingly, we present data from 199 patients, 98 in the placebo group and 101 patients in the tocilizumab group. Baseline characteristics are summarised in table 1.

**Table 1:** Baseline characteristics stratified by treatment allocation

	Tocilizumab (n=101)	Placebo (n=98)
Demographics		
Age, years	62±10	60±9
Men	80 (79)	87 (89)
Body mass index, kg/m^2^*	27.1±4.5	27.5±4.3
White	99 (98)	94 (96)
Smoking status		
Never smokers	38 (38)	36 (37)
Previous smokers	33 (33)	24 (24)
Current smokers	30 (30)	38 (39)
Prior conditions		
Diabetes mellitus	8 (8)	6 (6)
Hypertension	33 (33)	30 (31)
Treatment		
ACE inhibitor or ARB	22 (22)	25 (26)
Aldosterone antagonist	1 (1)	0 (0)
Oral anticoagulants	5 (5)	2 (2)
Platelet inhibitor	12 (12)	5 (5)
Beta-blocker	8 (8)	3 (3)
Calcium antagonist	13 (13)	10 (10)
Diuretic	8 (8)	8 (8)
Statin	19 (19)	9 (9)
Upfront DAPT	101 (100)	98 (100)
Clinical characteristics		
Systolic blood pressure at admission, systolic mm Hg*	131±23	132±22
Diastolic blood pressure at admission, mm Hg*	81±17	84±16
Time from symptom onset to arrival at PCI centre, minutes*	151±71	149±72
Door-to-balloon time, min*	23±10	23±11
GRACE risk score*	140±25	135±21
Blood samples		
Troponin T, ng/L†	44 (22–163)	49 (28–95)
Neutrophils, ×10^9^/L†	8 (6–11)	8 (6–10)
CRP, mg/L†	3 (1–5)	3 (1–5)
Infarct location		
Left anterior descending branch	38 (38)	36 (37)
Circumflex or marginal	11 (11)	13 (13)
Right coronary artery	47 (47)	46 (49)
Other	5 (5)	3 (3)

Values are n (%).

*Mean±SD.

†Median (IQR).

ARB, angiotensin receptor; CK-MB, creatine kinase myocardial band; CRP, C reactive protein; DAPT, dual antiplatelet therapy; HDL, high-density lipoprotein; LDL, low-density lipoprotein; NT-proBNP, N-terminal pro-B-type natriuretic peptide; PCI, percutaneous coronary intervention.

### Adverse effects

Minor variations were observed in specific biochemical factors between the two treatment groups, indicating potential side effects associated with tocilizumab. However, no significant differences in serious adverse events were found (online supplemental table 6). Importantly, there were no significant differences in these biochemical factors between the two treatment groups at 3 and 6 months.^8^

10.1136/openhrt-2023-002301.supp1 Supplementary data

### Cytokine levels during hospitalisation

There was no difference in circulating cytokine levels at baseline between the two treatment arms. We observed two different patterns of cytokine changes after hospital admission. The levels of some cytokines initially fell, followed by a rise towards the end of the hospitalisation (eg, eotaxin, IL-9, IP-10, MIP1-β, PDGF, RANTES, TNF- α). The concentrations of other cytokines increased throughout the hospitalisation (eg, G-CSF, IFN-γ, IL-1b, IL-2, IL-6, IL-7, IL-13, MIP-1α) (online supplemental table 1, online supplemental figure 2).

There was a significant increase in levels of several (16 out of 23) cytokines in the tocilizumab group compared with placebo (p<0.05), but after Bonferroni correction, only IL-6, IL-8 and IL-1ra were significant (p<0.002). Further subanalyses were, therefore, restricted to these three cytokines (online supplemental table 1).

### IL-6, IL-8 and IL-1ra levels during hospitalisation and according to time from symptom onset

**Figure 1:**
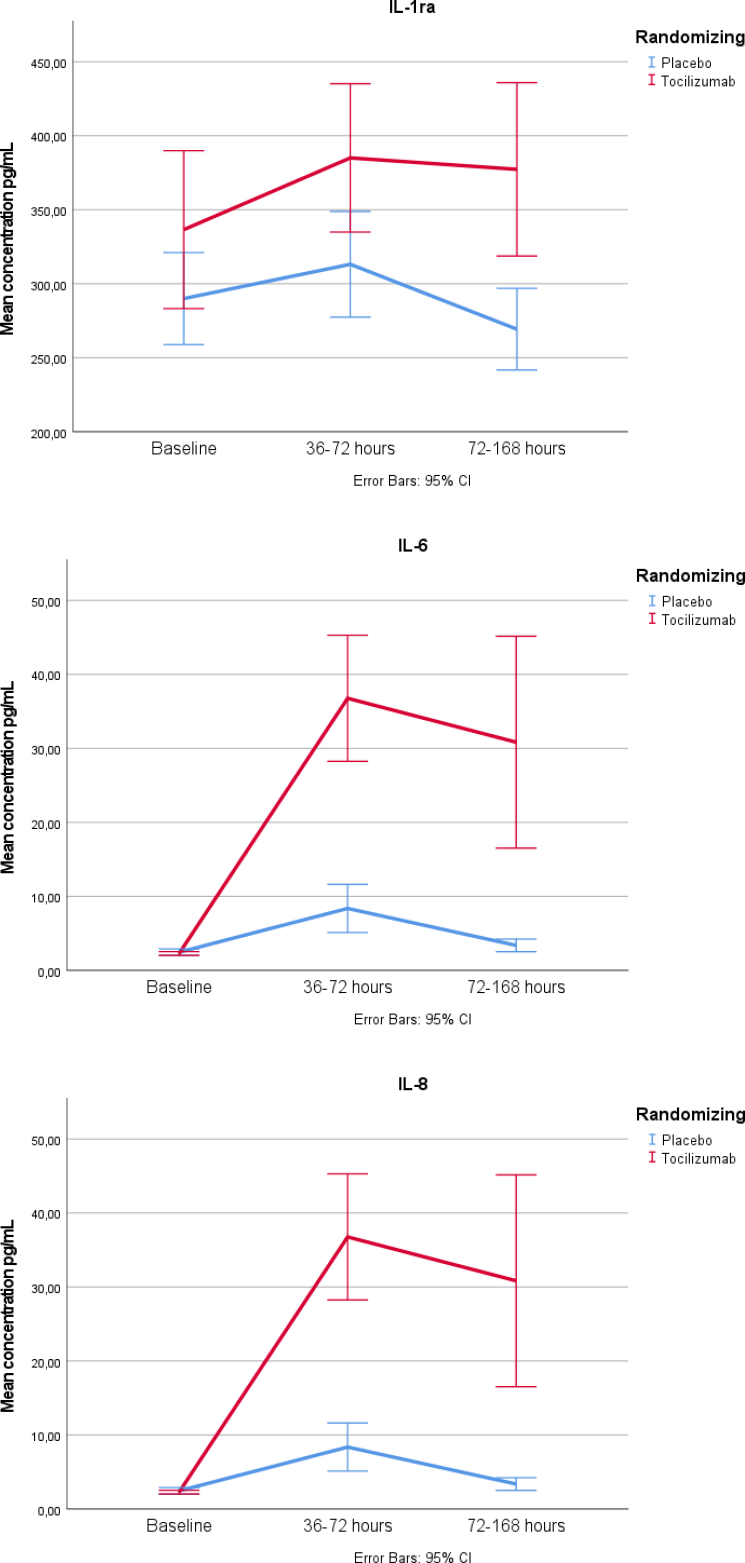
Mean and 95% CIs for cytokines with a significant group×time interaction after Bonferroni correction (p<0.002) during hospitalisation.

For IL-6, we observed a substantial increase in the tocilizumab group compared with both baseline and placebo during hospitalisation. A similar pattern was observed for IL-8 and IL-1ra (figure 1).

The increase in IL-6 in patients treated with tocilizumab was observed regardless of symptom duration, but the increase was more pronounced in the patients presenting >3 hours from symptom onset. For IL-1ra and IL-8, we only observed a significant treatment effect in those with symptom duration ≤3 hours (table 2).

**Table 2:** Multiplex cytokine assay during hospitalisation in patients with ST-elevation myocardial infarction receiving placebo (n=98) and tocilizumab (n=101), stratified by time (≤3 and >3 hours)

	Group	Baseline	36–72 hours	72–168 hours	P value
≤3 hours					
IL-1ra (pg/mL)	Placebo	289 (251–327)	330 (281–379)	271 (234–308)	0.005
	Tocilizumab	341 (275–406)	398 (328–468)	414 (329–499)†	
IL-6 (pg/mL)	Placebo	2.4 (2.1–2.7)	10 (6–15)	3.6 (2.4–4.7)	<0.001
	Tocilizumab	2.5 (2.2–2.8)	34 (26–42)‡	31 (11–51)‡	
IL-8 (pg/mL)	Placebo	2.9 (2.5–3.4)	3.3 (2.8–3.8)	2.3 (2.0–2.7)	0.001
	Tocilizumab	3 (2.5–3.4)	4.6 (3.8–5.3)†	4 (3.2–4.8)‡	
>3 hours					
IL-1ra (pg/mL)	Placebo	292 (236–348)	276 (236–316)	266 (227–305)	0.06
	Tocilizumab	328 (230–425)	358 (302–414)	301 (267–336)	
IL-6 (pg/mL)	Placebo	2.6 (1.3–3.9)	4.2 (3.4–5.1)	3 (2.1–3.9)	<0.001
	Tocilizumab	1.9 (1.5–2.4)	42 (21–64)‡	30 (15–45)‡	
IL-8 (pg/mL)	Placebo	2.5 (2–2.9)	3 (2.4–3.6)	2.4 (1.9–2.9)	0.04
	Tocilizumab	2.6 (2–3.2)	3.9 (3.1–4.6)	4 (2.4–5.7)*	

Data are mean and (95% CI). P value in table are mixed between-within subjects analysis of variance. P values in footnotes are between group differences in changes from baseline, independent sample t-test.

*p<0.05.

†p<0.01.

‡p<0.001.

IL-6, interleukin-6; IL-8, interleukin-8; IL-1ra, interleukin-1 receptor antagonist.

### Correlations between IL-6, IL-8 and IL-1ra and CMR, TnT and inflammatory markers

Interleukin-6_AUC_ and IL-8_AUC_ showed positive correlations with neutrophils_AUC_, CRP_AUC_ and TnT_AUC_ in both treatment groups. All these associations were more prominent in the placebo group for IL-8_AUC_, but only for neutrophils with IL-6_AUC_. Further, both IL-6_AUC_ and IL-8_AUC_ demonstrated a negative association with MSI and were associated with larger infarct sizes at admission and 6 months in the placebo group. The only exception was a moderate association between IL-6_AUC_ and infarct size at admission in the tocilizumab group. Interleukin-8_AUC_ showed a positive correlation to IL-6_AUC_ in both groups (table 3).

**Table 3:** Pearson correlations between IL-6_AUC_, IL-8_AUC_, IL1-ra_AUC_ and neutrophils_AUC,_ CRP_AUC,_ troponin T_AUC_, myocardial salvage index and infarct size (%)

	IL-6_AUC_						IL-8_AUC_						IL-1ra_AUC_					
	All		≤3 hours		>3 hours		All		≤3 hours		>3 hours		All		≤3 hours		>3 hours	
	Placebo	Toci	Placebo	Toci	Placebo	Toci	Placebo	Toci	Placebo	Toci	Placebo	Toci	Placebo	Toci	Placebo	Toci	Placebo	Toci
IL-6_AUC_	1	1	1	1	1	1	0.70†	0.48†	0.53†	0.39†	0.87†	0.63†	0.21*	0.14	0.07	0.17	0.34	0.17
Neutr_AUC_	0.73†	0.28†	0.35†	0.54†	0.93†	0.00	0.68†	0.27†	0.44†	0.38†	0.89†	0.01	0.33†	0.22*	0.16	0.28*	0.48†	0.01
CRP_AUC_	0.37†	0.36†	0.43†	0.05	0.48†	0.50†	0.58†	0.20*	0.70†	0.08	0.44*	0.33	0.09	0.02	0.09	0.06	0.13	0.02
TnT_AUC_	0.31†	0.29†	0.45†	0.33†	0.20	0.25	0.40†	0.27†	0.44†	0.31*	0.34	0.17	0.08	0.03	0.09	0.04	0.06	0.01
MSI	−0.22*	−0.18	−0.29*	−0.09	−0.17	−0.37*	−0.24*	−0.16	−0.25*	−0.14	−0.23	−0.22	−0.04	0.01	−0.06	0.00	−0.026	0.05
IS Adm	0.23*	0.21*	0.32†	0.16	0.15	0.33	0.29†	0.14	0.34†	0.17	0.19	0.06	0.05	−0.06	0.04	−0.03	0.085	−0.26
IS 6 months	0.25*	0.18	0.37†	0.16	0.14	0.26	0.34†	0.14	0.41†	0.19	0.22	0.01	0.08	−0.05	0.07	−0.03	0.11	−0.20

Data are shown for all patients and according to ≤3 or >3 hours from time of onset. Placebo n=98, tocilizumab n=101.

*p<0.05.

†p<0.001.

Adm, admission; AUC, area under the curve; CRP, C reactive protein; IL-6, interleukin-6; IL-8, interleukin-8; IL-1ra, interleukin-1 receptor antagonist; IS, infarct size; MSI, myocardial salvage index; Neutr, neutrophils; TnT, troponin T.

Interleukin-1ra_AUC_ showed weak positive correlation with neutrophils_AUC_ in both treatment arms, but there was no correlation with markers of cardiac injury (table 3).

### Correlations between IL-6, IL-8 and IL-1ra and CMR findings, TnT and inflammatory markers according to time from symptom onset

In the placebo group, both IL-6_AUC_ and IL-8_AUC_ correlated with neutrophils_AUC_ and CRP_AUC_, regardless of the time from symptom onset. However, the association with neutrophils_AUC_ was stronger in patients presenting >3 hours from symptom onset. This trend was also observed in the association between IL-6_AUC_ and IL-8_AUC_. The correlations of IL-6_AUC_ and IL-8_AUC_ with TnT_AUC_, MSI and infarct size were limited to patients presenting ≤3 hours from symptom onset (table 3).

In the tocilizumab group, both IL-6_AUC_ and IL-8_AUC_ showed a strong correlation with neutrophils_AUC_, but only in patients presenting ≤3 hours from symptom onset. Conversely, the association with CRP_AUC_ was limited to patients presenting >3 hours from symptom onset. There was a stronger correlation between IL-6_AUC_, IL-8_AUC_ and TNT_AUC_ in patients presenting ≤3 hours. Interleukin-6_AUC_ showed a negative correlation with MSI in patients arriving >3 hours from symptom onset, while this correlation was not observed for IL8_AUC_. There was no significant association between IL-6_AUC_ or IL-8_AUC_ and infarct size, regardless of the time from symptom onset. The positive correlation between IL-8_AUC_ and IL-6_AUC_ was observed regardless of the time since symptom onset (table 3).

In the placebo group, IL-1ra_AUC_ demonstrated an association with neutrophils in patients presenting >3 hours from symptom onset, while in the tocilizumab group, the association was observed in patients presenting ≤3 hours from symptom onset. There was no correlation between IL-1ra_AUC_ and markers of cardiac injury, irrespective of the time from symptom onset (table 3).

### Cytokine levels at 3–6 months

The concentration of all cytokines returned to baseline levels or lower at 3 and 6 months, and there were no significant differences between the treatment groups (online supplemental table 4).

## Discussion

We have previously reported that tocilizumab improves MSI in patients with STEMI.^8^ However, the mechanism for this beneficial effect is not yet clear. In this substudy of the ASSAIL-MI trial, we explored the effect of tocilizumab on 23 human cytokines.

### Main findings

The main findings were that (1) tocilizumab increased the concentrations of most cytokines in the acute phase compared with placebo; (2) a pronounced effect was observed for IL-6, IL-8 and IL-1ra also after adjusting for multiple comparisons and (3) the association between inflammation (IL-6 and IL-8) and myocardial injury (infarct size, TnT and MSI) was not observed in patients treated with tocilizumab. This association was primarily seen in placebo-treated patients who presented ≤3 hours from symptom onset.

### Interleukin-6

Elevated IL-6 has been demonstrated in ischaemic myocardium in rat models,^15^ in human myocardium following MI^16^ and has been described as an independent predictor of infarct size.^17^ As IL-6 is a major contributor to the inflammatory cascade and the subsequent reperfusion ischaemia injury, it has been hypothesised that IL-6 inhibition may be cardioprotective. Tocilizumab has shown to protect ischaemic cardiomyocytes by preventing apoptosis in ex vivo cell cultures^18^ providing further support for this hypothesis. There was a marked elevation in circulating levels of IL-6 in patients treated with tocilizumab. This well-known effect of tocilizumab treatment is caused by reduced clearance of IL-6 from the circulation due to inhibition of the cellular and soluble IL-6 receptors with tocilizumab, preventing IL-6 from binding.^19^ Even if tocilizumab increases the circulating levels of IL-6, RNA sequencing has shown a reduction in the signalling pathways when compared with placebo.^12^ This corresponds to the observed suppression of CRP in the tocilizumab group (online supplemental figure 1), which is shown to be a surrogate marker for sufficient tocilizumab levels to inhibit the effects of IL-6.^20^

We observed a significant correlation between IL-6 and TnT levels in both the tocilizumab and placebo group. In the group presenting ≤3 hours from symptom onset, IL-6 was associated with higher TnT levels, larger infarct sizes and less myocardial salvage in the placebo group. These associations were markedly attenuated in the tocilizumab group, suggesting that IL-6 may be an important therapeutic target in STEMI patients. This parallels findings with regard to procedure-related myocardial injury in NSTEMI.^7^

Interleukin-6 had a more pronounced increase in patients treated with tocilizumab presenting >3 hours from symptom onset that also correlated with lower MSI. This does not necessarily imply a negative effect of tocilizumab, but may reflect that upstream mediators of IL-6 release have already been activated. The further increase in IL-6 after the administration of tocilizumab occurs in a setting where IL-6 is uncoupled from its downstream effect. Indeed, patients presenting >3 hours from symptom onset in the ASSAIL-MI study had better effect of tocilizumab on MSI despite the reported increase in IL-6.

### Interleukin 8

IL-8 is associated with a worse clinical outcome in STEMI.^21^ As far as we know, no studies have addressed the effect of tocilizumab on IL-8 in MI, but it has been observed that tocilizumab is a potent inhibitor of IL-8 in triple-negative breast cancer.^22^ In this study, however, we showed a significant increase in IL-8 levels in the tocilizumab group, particularly in those presenting ≤3 hours from symptom onset. Moreover, in the placebo group, elevated IL-8 levels were associated with higher TnT, lower MSI and larger infarct size, restricted to patients presenting ≤3 hours from symptom onset. These associations were markedly attenuated in the tocilizumab group, reaching statistical significance only for TnT. The reason for this pattern is not clear, but one could hypothesise that even if tocilizumab increases IL-8 levels, it may attenuate IL-8 signalling at the cellular level like what has been observed with IL-6.^12^

### Interleukin-1ra

We observed a positive association between IL-1ra and neutrophils, but not CRP and no associations with markers of cardiac injury. The significant increase in IL-1ra in the tocilizumab treated patients could nevertheless be of relevance. Although no effect on the upstream cytokine IL-1b was found, an attenuating effect of IL-1 may be present as IL-1ra blocks the binding of both IL-1α and IL-1β to their common receptor.^23^ Although there were no correlations with outcomes, the increase in this anti-inflammatory mediator could be of relevance for the beneficial effects of tocilizumab in STEMI patients. In fact, the recombinant human IL-1ra anakinra have shown some beneficial effects in MI patients.^24 25^

### Neutrophils

We have recently showed a decrease in the number and function of neutrophils that were related to the beneficial effects of tocilizumab in the ASSAIL-MI population.^12^ In this study, we showed that IL-6 and IL-8 strongly correlated to the number of neutrophils, particularly in the placebo group. We have previously shown a similar pattern in NSTEMI, where strong positive correlations between neutrophil counts and acute phase proteins such as LPS binding protein, proteinase 3 and hepcidin were present in placebo-treated patients only.^26^ The attenuation of these associations in the tocilizumab group is consistent with an attenuating effect of IL-6 antagonism on the acute phase response and a plausible explanation for tocilizumab’s cardioprotective effect in acute coronary syndrome.

## Limitations

The sample size was estimated with regard to the endpoint MSI measured by CMR. As the circulating cytokine concentrations are very low, many of the differences are numerically small and our sample has reduced power to detect subtle effects on clinical endpoints. Due to limitations of the analysis kits, many results had to be recalculated manually, and thus, it may be difficult to reproduce absolute concentrations. Moreover, correlations do not necessarily imply causal relationship, particularly when multiple correlation analyses are performed. We have, however, corrected for multiple comparisons.

## Conclusion

Tocilizumab results in a significant increase in circulating levels of IL-6, IL-8 and IL-1ra in STEMI. Interleukin-6 and IL-8 showed correlations to neutrophils/CRP and markers of myocardial injury in the placebo group, which was attenuated in the tocilizumab group, potentially reflecting the beneficial effects of tocilizumab in the ischaemia-reperfusion injury in STEMI. Nonetheless, the rise in inflammatory mediators following tocilizumab treatment in STEMI may be seen as counterintuitive with regard to the observed beneficial effects. This may suggest that part of the effect is related to the inhibition of IL-6 mediated intracellular signalling, rather than secondary effects of other cytokines, as we have recently suggested for the effects of tocilizumab on neutrophil function in the same patients.

## Data Availability

Data are available on reasonable request.

## References

[1] Geovanini GR, Libby P. Atherosclerosis and inflammation: overview and updates. Clin Sci 2018;132:1243–52. 10.1042/CS20180306 29930142

[2] Ong S-B, Hernández-Reséndiz S, Crespo-Avilan GE, et al. Inflammation following acute myocardial infarction: multiple players, dynamic roles, and novel therapeutic opportunities. Pharmacol Ther 2018;186:73–87. 10.1016/j.pharmthera.2018.01.001 29330085 PMC5981007

[3] Ridker PM, Rane M. Interleukin-6 signaling and anti-interleukin-6 therapeutics in cardiovascular disease. Circ Res 2021;128:1728–46. 10.1161/CIRCRESAHA.121.319077 33998272

[4] Sheikh AS, Yahya S, Sheikh NS, et al. C-reactive protein as a predictor of adverse outcome in patients with acute coronary syndrome. Heart Views 2012;13:7–12. 10.4103/1995-705X.96660 22754634 PMC3385197

[5] Ridker PM. C-reactive protein and the prediction of cardiovascular events among those at intermediate risk: moving an inflammatory hypothesis toward consensus. J Am Coll Cardiol 2007;49:2129–38. 10.1016/j.jacc.2007.02.052 17531663

[6] Ridker PM, Everett BM, Thuren T, et al. Antiinflammatory therapy with Canakinumab for Atherosclerotic disease. N Engl J Med 2017;377:1119–31. 10.1056/NEJMoa1707914 28845751

[7] Kleveland O, Kunszt G, Bratlie M, et al. Effect of a single dose of the Interleukin-6 receptor antagonist Tocilizumab on inflammation and troponin T release in patients with non-ST-elevation myocardial infarction: a double-blind, randomized, placebo-controlled phase 2 trial. Eur Heart J 2016;37:2406–13. 10.1093/eurheartj/ehw171 27161611

[8] Broch K, Anstensrud AK, Woxholt S, et al. Randomized trial of Interleukin-6 receptor inhibition in patients with acute ST-segment elevation myocardial infarction. J Am Coll Cardiol 2021;77:1845–55. 10.1016/j.jacc.2021.02.049 33858620

[9] Kleveland O, Ueland T, Kunszt G, et al. Interleukin-6 receptor inhibition with Tocilizumab induces a selective and substantial increase in plasma IP-10 and MIP-1Β in non-ST-elevation myocardial infarction. Int J Cardiol 2018;271:1–7. 10.1016/j.ijcard.2018.04.136 29961572

[10] Di Stefano R, Di Bello V, Barsotti MC, et al. Inflammatory markers and cardiac function in acute coronary syndrome: difference in ST-segment elevation myocardial infarction (STEMI) and in non-STEMI models. Biomed Pharmacother 2009;63:773–80. 10.1016/j.biopha.2009.06.004 19906505

[11] Hjort M, Eggers KM, Lindhagen L, et al. Differences in biomarker concentrations and predictions of long-term outcome in patients with ST-elevation and non-ST-elevation myocardial infarction. Clin Biochem 2021;98:17–23. 10.1016/j.clinbiochem.2021.09.001 34496288

[12] Huse C, Anstensrud AK, Michelsen AE, et al. Interleukin-6 inhibition in ST-elevation myocardial infarction: immune cell profile in the randomised ASSAIL-MI trial. EBioMedicine 2022;80:104013. 10.1016/j.ebiom.2022.104013 35504178 PMC9079006

[13] Smolen JS, Beaulieu A, Rubbert-Roth A, et al. Effect of Interleukin-6 receptor inhibition with Tocilizumab in patients with rheumatoid arthritis (OPTION study): a double-blind, placebo-controlled, randomised trial lancet. Lancet 2008;371:987–97. 10.1016/S0140-6736(08)60453-5 18358926

[14] Heiberg E, Sjögren J, Ugander M, et al. Design and validation of segment--freely available software for cardiovascular image analysis. BMC Med Imaging 2010;10:1. 10.1186/1471-2342-10-1 20064248 PMC2822815

[15] Zhang J, Wang Q, Xue F, et al. Ischemic preconditioning produces more powerful anti-inflammatory and cardioprotective effects than limb remote ischemic postconditioning in rats with myocardial ischemia-reperfusion injury. Chin Med J (Engl) 2013;126:3949–55. 24157164

[16] Kanda T, Kobayashi I, Nagai R. Cardiac Interleukin-6 in ischemic myocardium. Circulation 2000;101:E86. 10.1161/01.cir.101.8.e86 10694537

[17] Tøllefsen IM, Shetelig C, Seljeflot I, et al. High levels of Interleukin-6 are associated with final infarct size and adverse clinical events in patients with STEMI. Open Heart 2021;8:e001869. 10.1136/openhrt-2021-001869 34933964 PMC8693166

[18] Cheng H-F, Feng Y, Jiang D-M, et al. Protective function of Tocilizumab in human cardiac myocytes ischemia reperfusion injury. Asian Pac J Trop Med 2015;8:48–52. 10.1016/S1995-7645(14)60186-3 25901924

[19] Oldfield V, Dhillon S, Plosker GL. Tocilizumab. Drugs 2009;69:609–32. 10.2165/00003495-200969050-00007 19368420

[20] Nishimoto N, Terao K, Mima T, et al. Mechanisms and pathologic significances in increase in serum Interleukin-6 (IL-6) and soluble IL-6 receptor after administration of an anti–IL-6 receptor antibody, Tocilizumab, in patients with rheumatoid arthritis and Castleman disease. Blood 2008;112:3959–64. 10.1182/blood-2008-05-155846 18784373

[21] Shetelig C, Limalanathan S, Hoffmann P, et al. Association of IL-8 with infarct size and clinical outcomes in patients with STEMI. J Am Coll Cardiol 2018;72:187–98. 10.1016/j.jacc.2018.04.053 29976293

[22] Alraouji NN, Aboussekhra A. Tocilizumab inhibits IL-8 and the proangiogenic potential of triple negative breast cancer cells. Mol Carcinog 2021;60:51–9. 10.1002/mc.23270 33264466

[23] Granowitz EV, Clark BD, Vannier E, et al. Effect of Interleukin-1 (IL-1) blockade on cytokine synthesis: I. IL-1 receptor antagonist inhibits IL-1-induced cytokine synthesis and blocks the binding of IL-1 to its type II receptor on human monocytes. Blood 1992;79:2356–63. 1533322

[24] Abbate A, Trankle CR, Buckley LF, et al. Interleukin-1 blockade inhibits the acute inflammatory response in patients with ST-segment-elevation myocardial infarction. J Am Heart Assoc 2020;9:e014941. 10.1161/JAHA.119.014941 32122219 PMC7335541

[25] Abbate A, Wohlford GF, Del Buono MG, et al. Interleukin-1 blockade with Anakinra and heart failure following ST-segment elevation myocardial infarction: results from a pooled analysis of the VCUART clinical trials. Eur Heart J Cardiovasc Pharmacother 2022;8:503–10. 10.1093/ehjcvp/pvab075 34617567 PMC9366639

[26] George MJ, Kleveland O, Garcia-Hernandez J, et al. Novel insights into the effects of interleukin 6 antagonism in non-ST-segment-elevation myocardial infarction employing the Somascan Proteomics platform. J Am Heart Assoc 2020;9:e015628. 10.1161/JAHA.119.015628 32515246 PMC7429051

