# Supplementary material for: Cytokine pattern in patients with ST-elevation myocardial infarction treated with the interleukin-6 receptor antagonist tocilizumab
Source: Open Heart. 2023 Aug 17;10(2):e002301. doi: 10.1136/openhrt-2023-002301 (PMC10441101; doi:10.1136/openhrt-2023-002301)
Supplement: Supplementary data [file openhrt-2023-002301supp001.pdf]

Supplemental Appendix

Cytokine pattern in patients with ST-elevation myocardial infarction treated with the interleukin-6 receptor antagonist Tocilizumab

Page 2
Supplemental table 1: Multiplex cytokine assay during hospitalisation in patients with ST-elevation myocardial infarction receiving placebo (n=98) and tocilizumab (n=101).

Page 3
Supplemental table 2: Multiplex cytokine assay during hospitalisation in patients with ST-elevation myocardial infarction receiving placebo (n=98) and tocilizumab (n=101) stratified by time (≤ 3 hours)

Page 4
Supplemental table 3: Multiplex cytokine assay during hospitalisation in patients with ST-elevation myocardial infarction receiving placebo (n=98) and tocilizumab (n=101) stratified by time (> 3 hours)

Page 5
Supplemental table 4: Multiplex cytokine assay at 3 and 6 months in patients with ST-elevation myocardial infarction receiving placebo (n=98) and tocilizumab (n=101).

Page 6
Supplemental table 5: Pearson correlations between IL-6_{AUC}, IL-8_{AUC}, IL1-ra_{AUC} and $neutrophils_{AUC}$, $lymphocytes_{AUC}$, $monocytes_{AUC}$, $eosinophils_{AUC}$ and $basophils_{AUC}$. Data are shown for all patients and according to ≤ or > 3 hours from time of onset. Placebo n = 98, Tocilizumab n = 101.

Page 7
Supplemental table 6: Number of patients with Serious Adverse Events and Events of Special Interest at 6 months follow-up.

Page 8
Supplemental figure 1: Mean and 95% confidence intervals for CRP during hospitalisation.

Page 9-18
Supplemental figure 2: Mean and 95% confidence intervals for all cytokines without a significant *group*time* interaction after Bonferroni correction (p>0.002) during hospitalisation.

Supplemental table 1: Multiplex cytokine assay during hospitalisation in patients with ST-elevation myocardial infarction receiving placebo (n=98) and tocilizumab (n=101).

	Group	Baseline	16-36 hours	72-168 hours	p
**Eotaxin (pg/ml)**	Placebo	27 (24-30)	15 (13-16)	17 (15-18)	0.038
	Tocilizumab	26 (23-29)	18 (16-19)	21 (19-23)	
**G-CSF (pg/ml)**	Placebo	22 (19-25)	22 (19-25)	23 (21-25)	0.003
	Tocilizumab	24 (20-28)	31 (27-35)	37 (30-43)	
**GM-CSF (pg/ml)**	Placebo	2.9 (2.7-3.1)	3.2 (2.9-3.6)	2.9 (2.7-3.2)	0.008
	Tocilizumab	3.3 (2.9-3.7)	3.9 (3.3-4.4)	3.9 (3.3-4.6)	
**IFN-γ (pg/ml)**	Placebo	5.3 (4.9-5.7)	5.7 (5.3-6.1)	5.6 (5.3-5.9)	0.006
	Tocilizumab	6.4 (5.5-7.4)	7 (6.2-7.8)	7 (5.8-8.2)	
**IL-1β (pg/ml)**	Placebo	0.9 (0.7-1.1)	1.3 (1.1-1.5)	1.2 (1-1.3)	0.01
	Tocilizumab	1 (0.9-1.1)	1.8 (1.4-2.2)	1.7 (1.3-2.2)	
**IL-1ra (pg/ml)**	Placebo	289 (259-320)	314 (279-349)	269 (242-297)	**<0.001**
	Tocilizumab	335 (285-386)	379 (331-427)	376 (318-434)**	
**IL-2 (pg/ml)**	Placebo	8.5 (8.3-8.7)	9 (8.7-9.3)	8.8 (8.5-9)	0.022
	Tocilizumab	8.7 (8.3-9.1)	9.4 (9-9.7)	9.6 (9.1-10.2)	
**IL-4 (pg/ml)**	Placebo	0.91 (0.85-0.96)	0.81 (0.77-0.86)	0.8 (0.77-0.84)	0.011
	Tocilizumab	0.95 (0.89-1.01)	0.9 (0.84-0.96)	0.96 (0.89-1.03)	
**IL-5 (pg/ml)**	Placebo	36 (33-39)	31 (29-34)	32 (29-35)	0.005
	Tocilizumab	44 (36-52)	42 (36-47)	52 (27-77)	
**IL-6 (pg/ml)**	Placebo	2.4 (2-2.9)	9.3 (5.6-12.9)	3.4 (2.5-4.2)	**<0.001**
	Tocilizumab	2.8 (1.8-3.8)	37.1 (28.8-45.3)***	30.9 (16.7-45)***	
**IL-7 (pg/ml)**	Placebo	13 (11-14)	22 (20-24)	19 (18-21)	0.575
	Tocilizumab	13 (12-14)	22 (20-23)	20 (17-24)	
**IL-8 (pg/ml)**	Placebo	2.8 (2.5-3.1)	3.3 (2.9-3.7)	2.3 (2.1-2.6)	**<0.001**
	Tocilizumab	3 (2.6-3.4)	4.3 (3.8-4.9)***	4 (3.2-4.7)***	
**IL-9 (pg/ml)**	Placebo	157 (137-176)	114 (98-130)	171 (157-184)	0.461
	Tocilizumab	165 (145-185)	123 (107-139)	177 (163-192)	
**IL-12(p70) (pg/ml)**	Placebo	1.5 (1.3-1.7)	1.7 (1.5-1.9)	1.7 (1.5-1.9)	0.227
	Tocilizumab	2.3 (1.5-3.1)	2.1 (1.8-2.4)	2.9 (1.1-4.8)	
**IL-13 (pg/ml)**	Placebo	1 (0.7-1.4)	2.8 (2.2-3.4)	2 (1.5-2.4)	0.003
	Tocilizumab	1.6 (1-2.1)	3.7 (3-4.4)	3.4 (2.7-4.1)	
**IP-10 (pg/ml)**	Placebo	292 (243-342)	142 (119-164)	190 (162-219)	0.018
	Tocilizumab	290 (219-361)	181 (152-210)	307 (219-394)	
**MCP-1 (pg/ml)**	Placebo	30 (26-33)	15 (14-17)	11 (10-12)	0.006
	Tocilizumab	26 (23-29)	19 (17-22)	16 (14-18)	
**MIP-1α (pg/ml)**	Placebo	1.11 (0.98-1.25)	1.12 (1.01-1.22)	1.23 (1.13-1.32)	0.025
	Tocilizumab	1.2 (1.01-1.38)	1.34 (1.2-1.49)	1.58 (1.36-1.8)	
**MIP-1β (pg/ml)**	Placebo	100 (90-110)	76 (67-85)	109 (102-117)	0.564
	Tocilizumab	102 (92-112)	81 (72-90)	110 (102-118)	
**PDGF-bb (pg/ml)**	Placebo	168 (150-185)	125 (114-136)	137 (129-144)	0.413
	Tocilizumab	162 (145-179)	123 (117-130)	136 (125-146)	
**Rantes (pg/ml)**	Placebo	3654 (3014-4293)	1355 (1045-1665)	2385 (2070-2700)	0.675
	Tocilizumab	3640 (3027-4254)	1460 (1157-1762)	2457 (2124-2790)	
**TNF-α (pg/ml)**	Placebo	19 (17-21)	14 (13-16)	19 (17-21)	0.045
	Tocilizumab	21 (17-24)	17 (15-19)	24 (20-28)	
**VEGF (pg/ml)**	Placebo	75 (73-77)	69 (68-71)	68 (67-69)	0.07
	Tocilizumab	75 (73-78)	73 (70-75)	73 (70-76)	

Data are mean and (95% confidence interval). p, mixed between-within subjects analysis of variance. *p<0.05, **p<0.01, ***p<0.001, between group differences in changes from baseline, independent sample t-test. Significant p values after Bonferroni correction (p<0.002) marked as bold.

G-CSF = Granulocyte colony stimulating factor. GM-CSF = granulocyte-macrophage colony-stimulating factor. IFN-γ = Interferon-gamma. IL = Interleukin. ra = receptor antagonist. IP = interferon-γ inducible protein. MCP = macrophage chemoattractant protein. MIP = macrophage inflammatory protein. PDGF = platelet-derived growth factor. RANTES = regulated on activation, normal T cell expressed and secreted. TNF = tumor necrosis factor. VEGF = Vascular endothelial growth factor.

Woxholt S, *et al. Open Heart* 2023; 10:e002301. doi: 10.1136/openhrt-2023-002301

Supplemental table 2: Multiplex cytokine assay during hospitalisation in patients with ST-elevation myocardial infarction receiving placebo (n=98) and tocilizumab (n=101) stratified by time (≤ 3 hours)

	Group	Baseline	36-72 hours	72-168 hours	P
**Eotaxin (pg/ml)**	Placebo	27 (24-31)	15 (13-16)	17 (15-19)	0.071
	Tocilizumab	27 (23-32)	18 (16-20)	20 (18-22)	
**G-CSF (pg/ml)**	Placebo	23 (19-26)	22 (18-25)	22 (19-25)	0.09
	Tocilizumab	24 (20-27)	30 (25-34)	38 (29-48)	
**GM-CSF (pg/ml)**	Placebo	2.9 (2.7-3.2)	3.4 (2.9-3.9)	3 (2.7-3.3)	0.047
	Tocilizumab	3.3 (2.8-3.9)	4.1 (3.3-4.9)	4.3 (3.3-5.3)*	
**IFN-γ (pg/ml)**	Placebo	5.4 (4.9-6)	5.9 (5.4-6.4)	5.7 (5.3-6)	0.021
	Tocilizumab	6.2 (5.3-7)	6.7 (6-7.5)	7.6 (5.8-9.3)	
**IL-1β (pg/ml)**	Placebo	1 (0.7-1.2)	1.4 (1.1-1.6)	1.2 (1-1.3)	0.095
	Tocilizumab	0.9 (0.8-1)	1.6 (1.3-1.9)	1.8 (1.1-2.5)	
**IL-1ra (pg/ml)**	Placebo	289 (251-327)	330 (281-379)	271 (234-308)	0.005
	Tocilizumab	341 (275-406)	398 (328-468)	414 (329-499)**	
**IL-2 (pg/ml)**	Placebo	8.6 (8.3-8.9)	9.2 (8.8-9.6)	8.8 (8.5-9.2)	0.14
	Tocilizumab	8.7 (8.1-9.2)	9.4 (9-9.9)	9.9 (9.1-10.7)	
**IL-4 (pg/ml)**	Placebo	0.9 (0.9-1)	0.8 (0.8-0.9)	0.8 (0.8-0.8)	0.026
	Tocilizumab	1 (0.9-1)	0.9 (0.8-1)	1 (0.9-1.1)**	
**IL-5 (pg/ml)**	Placebo	36 (33-39)	32 (29-35)	31 (28-34)	0.003
	Tocilizumab	40 (35-45)	39 (36-43)*	59 (22-96)**	
**IL-6 (pg/ml)**	Placebo	2.4 (2.1-2.7)	10 (6-15)	3.6 (2.4-4.7)	**<0.001**
	Tocilizumab	2.5 (2.2-2.8)	34 (26-42)***	31 (11-51)***	
**IL-7 (pg/ml)**	Placebo	13 (11-14)	22 (20-24)	19 (17-21)	0.49
	Tocilizumab	13 (12-14)	22 (20-23)	21 (17-26)	
**IL-8 (pg/ml)**	Placebo	2.9 (2.5-3.4)	3.3 (2.8-3.8)	2.3 (2-2.7)	**0.001**
	Tocilizumab	3 (2.5-3.4)	4.6 (3.8-5.3)**	4 (3.2-4.8)***	
**IL-9 (pg/ml)**	Placebo	156 (134-178)	121 (103-140)	166 (152-181)	0.382
	Tocilizumab	173 (147-200)	121 (101-142)	177 (158-196)	
**IL-12(p70) (pg/ml)**	Placebo	1.6 (1.3-1.8)	1.8 (1.5-2)	1.7 (1.4-1.9)	0.393
	Tocilizumab	2 (1.5-2.5)	2 (1.6-2.4)	3.4 (0.7-6.2)	
**IL-13 (pg/ml)**	Placebo	1.2 (0.7-1.7)	3.1 (2.3-3.8)	2.1 (1.5-2.7)	0.02
	Tocilizumab	1.7 (0.9-2.6)	4.1 (3.1-5.1)	3.8 (2.8-4.8)	
**IP-10 (pg/ml)**	Placebo	295 (229-360)	137 (112-162)	190 (151-229)	0.013
	Tocilizumab	290 (215-365)	181 (144-219)*	336 (205-466)***	
**MCP-1 (pg/ml)**	Placebo	31 (27-36)	15 (13-17)	11 (10-12)	0.017
	Tocilizumab	27 (23-30)	19 (17-22)***	16 (14-18)***	
**MIP-1α (pg/ml)**	Placebo	1.1 (1-1.3)	1.1 (1-1.3)	1.2 (1.1-1.3)	0.01
	Tocilizumab	1.2 (1-1.3)	1.3 (1.1-1.5)	1.7 (1.3-2)**	
**MIP-1β (pg/ml)**	Placebo	101 (88-114)	81 (70-92)	108 (99-117)	0.808
	Tocilizumab	105 (93-118)	78 (67-89)	110 (100-121)	
**PDGF-bb (pg/ml)**	Placebo	168 (147-189)	127 (111-143)	133 (125-142)	0.666
	Tocilizumab	161 (144-178)	119 (112-127)	137 (123-152)	
**Rantes (pg/ml)**	Placebo	3507 (2740-4274)	1362 (1019-1704)	2230 (1868-2592)	0.702
	Tocilizumab	3748 (2984-4512)	1383 (1000-1765)	2491 (2053-2928)	
**TNF-α (pg/ml)**	Placebo	17 (15-19)	14 (12-15)	17 (16-19)	0.041
	Tocilizumab	18 (16-20)	15 (14-17)	23 (18-28)	
**VEGF (pg/ml)**	Placebo	76 (74-77)	70 (68-72)	68 (66-70)	0.051
	Tocilizumab	75 (72-77)	73 (70-76)	75 (71-79)	

Data are mean and (95% confidence interval). p, mixed between-within subjects analysis of variance.
*p<0.05, **p<0.01, ***p<0.001, between group differences in changes from baseline, independent sample t-test. Significant p values after Bonferroni correction (p<0.002) marked as bold.
G-CSF = Granulocyte colony stimulating factor. GM-CSF = granulocyte-macrophage colony-stimulating factor. IFN-γ = Interferon-gamma. IL = Interleukin. ra = receptor antagonist. IP = interferon-γ inducible protein. MCP = macrophage chemoattractant protein. MIP = macrophage inflammatory protein. PDGF = platelet-derived growth factor. RANTES = regulated on activation, normal T cell expressed and secreted. TNF = tumor necrosis factor. VEGF = Vascular endothelial growth factor.

Woxholt S, *et al. Open Heart* 2023; 10:e002301. doi: 10.1136/openhrt-2023-002301

Supplemental table 3: Multiplex cytokine assay during hospitalisation in patients with ST-elevation myocardial infarction receiving placebo (n=98) and tocilizumab (n=101) stratified by time (> 3 hours)

	Group	Baseline	36-72 hours	72-168 hours	p
**Eotaxin (pg/ml)**	Placebo	26 (21-32)	16 (13-19)	16 (14-19)	0.316
	Tocilizumab	22 (19-25)	17 (14-19)	22 (18-26)	
**G-CSF (pg/ml)**	Placebo	23 (17-28)	19 (15-24)	25 (20-30)	0.138
	Tocilizumab	23 (12-34)	30 (23-38)	33 (26-40)	
**GM-CSF (pg/ml)**	Placebo	2.7 (2.5-3)	2.9 (2.6-3.2)	2.7 (2.4-3)	0.038
	Tocilizumab	3.2 (2.6-3.9)	3.4 (2.9-3.9)	3.2 (2.9-3.5)	
**IFN-γ (pg/ml)**	Placebo	5.1 (4.5-5.8)	5.3 (4.6-6)	5.3 (4.7-5.9)	0.125
	Tocilizumab	5.5 (4.5-6.4)	6.4 (5.6-7.1)	5.9 (5.2-6.6)	
**IL-1β (pg/ml)**	Placebo	0.8 (0.7-0.9)	1.2 (1-1.5)	1.2 (1-1.4)	0.029
	Tocilizumab	1.1 (0.8-1.4)	2.2 (0.9-3.4)	1.6 (1.2-2.1)	
**IL-1ra (pg/ml)**	Placebo	292 (236-348)	276 (236-316)	266 (227-305)	0.06
	Tocilizumab	328 (230-425)	358 (302-414)	301 (267-336)	
**IL-2 (pg/ml)**	Placebo	8.4 (8-8.8)	8.6 (8.1-9.1)	8.5 (8.1-8.9)	0.022
	Tocilizumab	8.7 (8.2-9.2)	9.3 (8.7-9.8)	9.1 (8.6-9.5)	
**IL-4 (pg/ml)**	Placebo	0.8 (0.7-0.9)	0.8 (0.7-0.8)	0.8 (0.7-0.9)	0.184
	Tocilizumab	0.8 (0.8-0.9)	0.8 (0.8-0.9)	0.9 (0.8-1)	
**IL-5 (pg/ml)**	Placebo	37 (30-45)	30 (24-37)	34 (26-42)	0.347
	Tocilizumab	42 (26-59)	38 (31-45)	37 (32-42)	
**IL-6 (pg/ml)**	Placebo	2.6 (1.3-3.9)	4.2 (3.4-5.1)	3 (2.1-3.9)	**<0.001**
	Tocilizumab	1.9 (1.5-2.4)	42 (21-64)***	30 (15-45)***	
**IL-7 (pg/ml)**	Placebo	13 (10-15)	23 (19-28)	19 (15-23)	1
	Tocilizumab	13 (11-15)	21 (18-24)	18 (16-20)	
**IL-8 (pg/ml)**	Placebo	2.5 (2-2.9)	3 (2.4-3.6)	2.4 (1.9-2.9)	0.04
	Tocilizumab	2.6 (2-3.2)	3.9 (3.1-4.6)	4 (2.4-5.7)*	
**IL-9 (pg/ml)**	Placebo	165 (125-205)	104 (70-137)	180 (151-209)	0.896
	Tocilizumab	152 (121-183)	128 (97-159)	175 (152-199)	
**IL-12(p70) (pg/ml)**	Placebo	1.3 (1-1.5)	1.5 (1.3-1.8)	1.8 (1.5-2.1)	0.347
	Tocilizumab	2.7 (0.3-5)	2.1 (1.6-2.6)	1.9 (1.5-2.3)	
**IL-13 (pg/ml)**	Placebo	0.7 (0.4-1.1)	2.3 (1.5-3)	1.7 (1.1-2.2)	0.062
	Tocilizumab	1.3 (0.6-1.9)	3.2 (2.1-4.2)	2.7 (1.9-3.4)	
**IP-10 (pg/ml)**	Placebo	293 (213-373)	153 (102-203)	191 (153-229)	0.696
	Tocilizumab	278 (100-457)	164 (115-213)	247 (198-297)	
**MCP-1 (pg/ml)**	Placebo	27 (19-34)	16 (13-19)	12 (10-14)	0.175
	Tocilizumab	23 (19-28)	19 (15-22)	17 (14-19)	
**MIP-1α (pg/ml)**	Placebo	1.1 (0.9-1.3)	1.1 (0.9-1.2)	1.3 (1.2-1.5)	0.759
	Tocilizumab	1.2 (0.6-1.8)	1.3 (1-1.5)	1.4 (1.2-1.6)	
**MIP-1β (pg/ml)**	Placebo	102 (82-121)	69 (51-86)	112 (97-127)	0.835
	Tocilizumab	95 (78-113)	85 (68-101)	108 (95-120)	
**PDGF-bb (pg/ml)**	Placebo	171 (139-203)	120 (110-131)	144 (129-158)	0.435
	Tocilizumab	148 (126-170)	130 (115-144)	131 (119-144)	
**Rantes (pg/ml)**	Placebo	4173 (2920-5426)	1415 (710-2119)	2726 (2094-3358)	0.862
	Tocilizumab	3421 (2306-4536)	1700 (1107-2293)	2370 (1837-2903)	
**TNF-α (pg/ml)**	Placebo	23 (18-27)	16 (12-21)	22 (19-26)	0.467
	Tocilizumab	26 (15-37)	20 (15-25)	26 (20-31)	
**VEGF (pg/ml)**	Placebo	74 (71-77)	69 (67-71)	68 (66-69)	0.858
	Tocilizumab	72 (70-74)	70 (67-73)	69 (67-71)	

Data are mean and (95% confidence interval). p, mixed between-within subjects analysis of variance.
*p<0.05, **p<0.01, ***p<0.001, between group differences in changes from baseline, independent sample t-test. Significant p values after Bonferroni correction (p<0.002) marked as bold.
G-CSF = Granulocyte colony stimulating factor. GM-CSF = granulocyte-macrophage colony-stimulating factor. IFN-γ = Interferon-gamma. IL = Interleukin. ra = receptor antagonist. IP = interferon-γ inducible protein. MCP = macrophage chemoattractant protein. MIP = macrophage inflammatory protein. PDGF = platelet-derived growth factor. RANTES = regulated on activation, normal T cell expressed and secreted. TNF = tumor necrosis factor. VEGF = Vascular endothelial growth factor.

Woxholt S, *et al. Open Heart* 2023; 10:e002301. doi: 10.1136/openhrt-2023-002301

Supplemental table 4: Multiplex cytokine assay at 3 and 6 months in patients with ST-elevation myocardial infarction receiving placebo (n=98) and tocilizumab (n=101).

	Group	Baseline	3 months	6 months	p
**Eotaxin (pg/ml)**	Placebo	27 (24-30)	18 (16-20)	17 (16-18)	0,815
	Tocilizumab	26 (23-29)	18 (16-20)	18 (17-20)	
**FGFb (pg/ml)**	Placebo	19,7 (19,6-19,8)	19,7 (19,6-19,8)	19,6 (19,5-19,7)	0,012
	Tocilizumab	20 (19,8-20,2)	20 (19,7-20,4)	20 (19,7-20,2)	
**G-CSF (pg/ml)**	Placebo	22 (19-25)	20 (17-23)	19 (17-21)	0,379
	Tocilizumab	24 (20-28)	22 (19-24)	22 (19-25)	
**GM-CSF (pg/ml)**	Placebo	2,9 (2,7-3,1)	2,7 (2,5-3)	2,5 (2,4-2,7)	0,07
	Tocilizumab	3,3 (2,9-3,7)	2,9 (2,6-3,2)	2,8 (2,6-3)	
**IFN-γ (pg/ml)**	Placebo	5,3 (4,9-5,7)	5 (4,7-5,4)	4,8 (4,6-5,1)	0,005
	Tocilizumab	6,4 (5,5-7,4)	6,4 (5,1-7,8)	6 (5,3-6,7)	
**IL-1β (pg/ml)**	Placebo	0,9 (0,7-1,1)	0,9 (0,8-1)	0,8 (0,7-0,9)	0,022
	Tocilizumab	1 (0,9-1,1)	1,2 (0,8-1,7)	1 (0,8-1,2)	
**IL-1ra (pg/ml)**	Placebo	289 (259-320)	235 (206-263)	222 (205-238)	0,023
	Tocilizumab	335 (285-386)	255 (223-286)	247 (224-270)	
**IL-2 (pg/ml)**	Placebo	8,5 (8,3-8,7)	8,5 (8,2-8,7)	8,4 (8,2-8,6)	0,089
	Tocilizumab	8,7 (8,3-9,1)	8,7 (8,3-9)	9,4 (7,9-11)	
**IL-4 (pg/ml)**	Placebo	0,91 (0,85-1)	0,75 (0,7-0,8)	0,72 (0,69-0,8)	0,019
	Tocilizumab	0,95 (0,89-1)	0,81 (0,76-0,9)	0,81 (0,76-0,9)	
**IL-5 (pg/ml)**	Placebo	36 (33-39)	31 (28-33)	30 (27-33)	0,176
	Tocilizumab	44 (36-52)	46 (22-70)	41 (29-54)	
**IL-6 (pg/ml)**	Placebo	2,4 (2-2,9)	1,5 (1,4-1,7)	1,5 (1,3-1,6)	0,226
	Tocilizumab	2,8 (1,8-3,8)	1,7 (1,5-1,9)	1,6 (1,5-1,8)	
**IL-7 (pg/ml)**	Placebo	13 (11-14)	18 (17-19)	16 (15-17)	0,158
	Tocilizumab	13 (12-14)	20 (14-26)	18 (17-20)	
**IL-8 (pg/ml)**	Placebo	2,8 (2,5-3,1)	1,8 (1,5-2)	1,7 (1,5-1,9)	0,059
	Tocilizumab	3 (2,6-3,4)	2,2 (1,9-2,5)	5,9 (-1,3-13,1)	
**IL-9 (pg/ml)**	Placebo	157 (137-176)	158 (143-174)	151 (138-165)	0,083
	Tocilizumab	165 (145-185)	173 (159-187)	171 (156-185)	
**IL-12(p70) (pg/ml)**	Placebo	1,5 (1,3-1,7)	1,5 (1,3-1,6)	1,3 (1,1-1,5)	0,004
	Tocilizumab	2,3 (1,5-3,1)	2,6 (1,1-4)	2,1 (1,5-2,6)	
**IL-13 (pg/ml)**	Placebo	1 (0,7-1,4)	1,1 (0,7-1,6)	0,9 (0,7-1,2)	0,003
	Tocilizumab	1,6 (1-2,1)	1,4 (1,1-1,6)	1,1 (0,9-1,3)	
**IL-15 (pg/ml)**	Placebo	356 (355-357)	351 (351-352)	351 (350-351)	0,256
	Tocilizumab	356 (355-357)	352 (351-354)	352 (351-353)	
**IP-10 (pg/ml)**	Placebo	292 (243-342)	205 (164-247)	188 (156-220)	0,852
	Tocilizumab	290 (219-361)	193 (167-220)	220 (166-274)	
**MCP-1 (pg/ml)**	Placebo	30 (26-33)	14 (12-15)	12 (11-13)	0,979
	Tocilizumab	26 (23-29)	14 (12-15)	13 (12-14)	
**MIP-1α (pg/ml)**	Placebo	1,11 (0,98-1,3)	1,14 (1,02-1,3)	1,08 (0,97-1,2)	0,275
	Tocilizumab	1,2 (1,01-1,4)	1,2 (1,11-1,3)	1,17 (1,07-1,3)	
**MIP-1β (pg/ml)**	Placebo	100 (90-110)	102 (93-110)	97 (90-105)	0,181
	Tocilizumab	102 (92-112)	107 (100-115)	106 (98-114)	
**PDGF-bb (pg/ml)**	Placebo	168 (150-185)	127 (117-136)	122 (116-128)	0,22
	Tocilizumab	162 (145-179)	130 (123-137)	134 (127-141)	
**Rantes (pg/ml)**	Placebo	3654 (3014-4293)	2265 (1909-2621)	2138 (1823-2453)	0,139
	Tocilizumab	3640 (3027-4254)	2431 (2099-2764)	2619 (2275-2963)	
**TNF-α (pg/ml)**	Placebo	19 (17-21)	17 (15-19)	17 (15-18)	0,025
	Tocilizumab	21 (17-24)	21 (18-24)	21 (18-24)	
**VEGF (pg/ml)**	Placebo	75 (73-77)	68 (66-69)	67 (66-68)	0,304
	Tocilizumab	75 (73-78)	69 (67-71)	69 (67-71)	

Data are mean and (95% confidence interval). p, mixed between-within subjects analysis of variance.
*p<0.05, **p<0.01, ***p<0.001, between group differences in changes from baseline, independent sample t-test. Significant p values after Bonferroni correction (p<0.002) marked as bold.
G-CSF = Granulocyte colony stimulating factor. GM-CSF = granulocyte-macrophage colony-stimulating factor. IFN-γ = Interferon-gamma. IL = Interleukin. ra = receptor antagonist. IP = interferon-γ inducible protein. MCP = macrophage chemoattractant protein. MIP = macrophage inflammatory protein. PDGF = platelet-derived growth factor. RANTES = regulated on activation, normal T cell expressed and secreted. TNF = tumor necrosis factor. VEGF = Vascular endothelial growth factor.

Supplemental table 5: Pearson correlations between IL-6_{AUC}, IL-8_{AUC}, IL1-ra_{AUC} and $neutrophils_{AUC}$, $lymphocytes_{AUC}$, $monocytes_{AUC}$, $eosinophils_{AUC}$ and $basophils_{AUC}$. Data are shown for all patients and according to ≤ or > 3 hours from time of onset. Placebo n = 98, Tocilizumab n = 101.

	**IL-6_{AUC}**						**IL-8_{AUC}**						**IL-1ra_{AUC}**					
	**All**		**≤ 3 h**		**> 3 h**		**All**		**≤ 3 h**		**> 3 h**		**All**		**≤ 3 h**		**> 3 h**	
	Placebo	Toci	Placebo	Toci	Placebo	Toci	Placebo	Toci	Placebo	Toci	Placebo	Toci	Placebo	Toci	Placebo	Toci	Placebo	Toci
**IL-6_{AUC}**	1	1	1	1	1	1	0.70**	0.48**	0.53**	0.39**	0.87**	0.63**	0.21*	0.14	0.07	0.17	0.34	0.17
**$Neutr_{AUC}$**	0.73**	0.28**	0.35**	0.54**	0.93**	0.00	0.68**	0.27**	0.44**	0.38**	0.89**	0.01	0.33**	0.22*	0.16	0.28*	0.48**	0.01
**$Lymph_{AUC}$**	0.04	0.00	0.02	0.10	0.11	-0.13	0.19	0.28**	0.176	0.35**	0.23	0.09	0.28**	0.32**	0.16	0.32**	0.49*	0.37*
**$Mono_{AUC}$**	0.01	0.11	0.00	-0.09	0.007	0.33	0.23*	0.16	0.268*	0.08	0.10	0.32	0.04	0.12	0.09	0.08	-0.05	0.28
**$Eosin_{AUC}$**	0.02	0.01	0.00	-0.14	-0.05	0.17	0.25*	0.09	0.291*	0.01	0.04	0.25	0.07	0.06	0.13	0.03	-0.09	0.24
**$Baso_{AUC}$**	-0.02	0.00	-0.02	-0.12	-0.01	0.16	0.25*	0.14	0.307*	0.09	0.16	0.27	0.1	0.12	0.15	0.09	0.04	0.31

IL = Interleukin. AUC = Area under the curve. Neutr = Neutrophils. Lymph = Lymphocytes. Mono = Monocytes. Eosin = Eosinophils. Baso = Basophils. Toci = Tocilisumab. h = hours.

Supplemental table 6: Number of patients with Serious Adverse Events and Events of Special Interest at 6 months follow-up.

**Adverse event**	**Tocilizumab**	**Placebo**
Any serious adverse event	19	15
Infections requiring hospitalization	3	2
New malignancy	2	0
Cardiovascular events	9	10
Myocardial infarction	0	4
CABG	1	0
Chest pain	5	4
Resuscitated VF	1	1
VT	1	0
Ischemic stroke	0	1
SAH	1	0
Worsening renal function*	0	0
Liver-associated events†	0	0

CABG = coronary artery bypass grafting; SAH = subarachnoid hemorrhage; VF = ventricular fibrillation; VT = ventricular tachycardia.

Source: This table is adapted from our previous publication: Broch K, Anstensrud AK, Woxholt S, et al. Randomized Trial of Interleukin-6 Receptor Inhibition in Patients With Acute ST-Segment Elevation Myocardial Infarction. Journal of the American College of Cardiology. 2021;77(15):1845-55.

Supplemental figure 1: Mean concentration and 95% confidence intervals for CRP during hospitalisation.

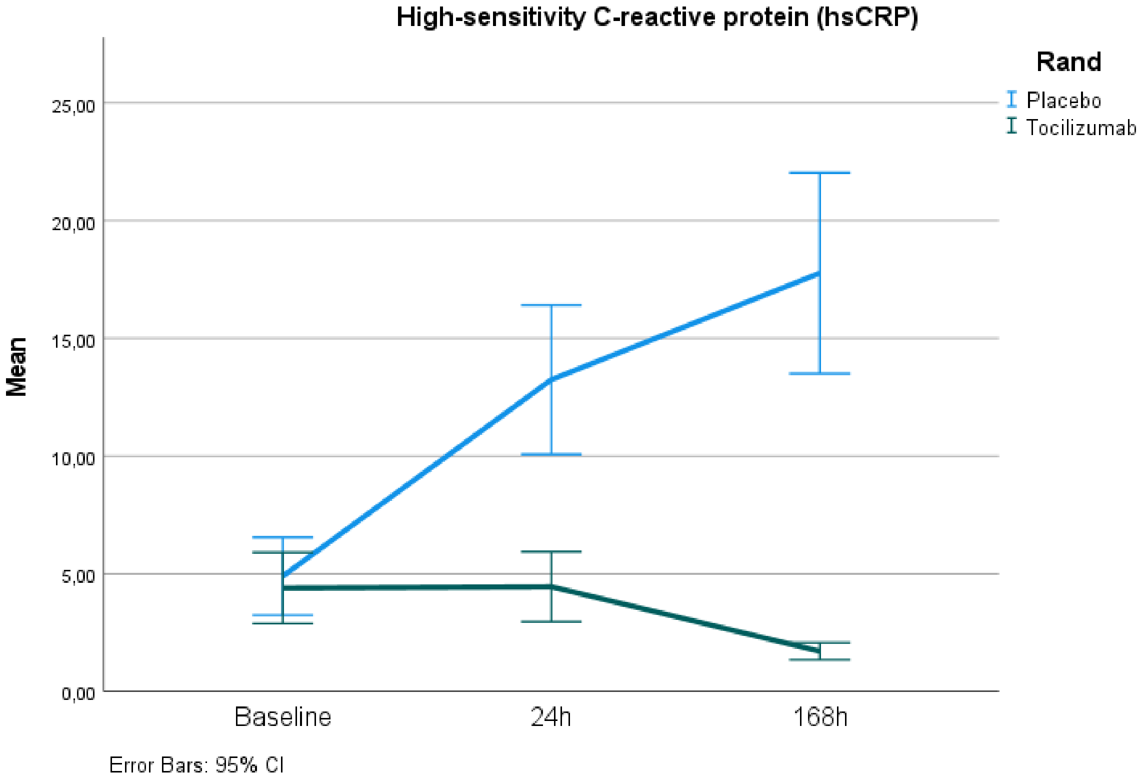

Woxholt S, et al. Open Heart 2023; 10:e002301. doi: 10.1136/openhrt-2023-002301

Supplemental figure 2: Mean and 95% confidence intervals for all cytokines without a significant *group*time* interaction after Bonferroni correction (p>0.002) during hospitalisation.

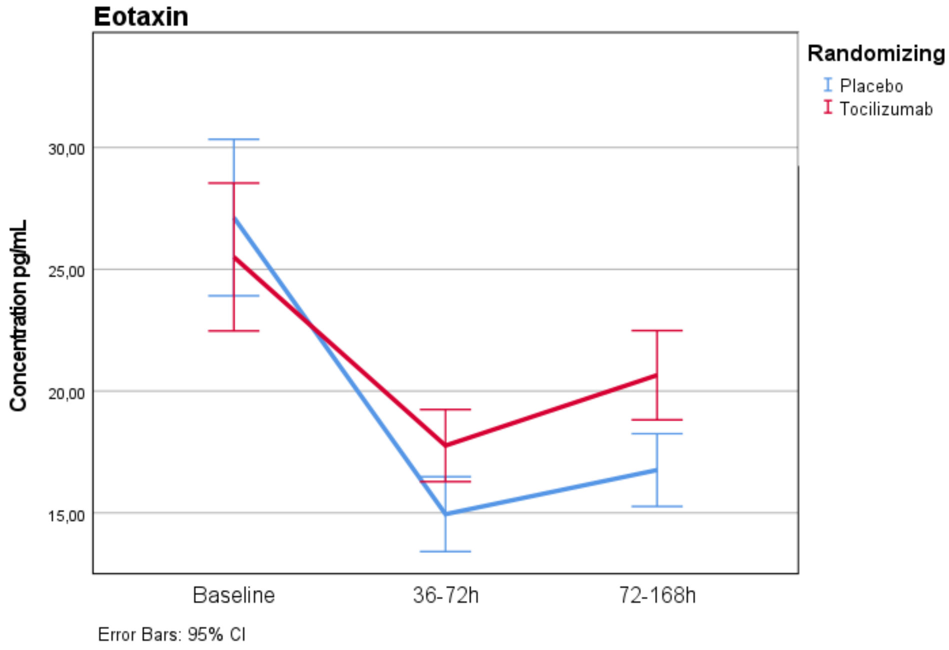

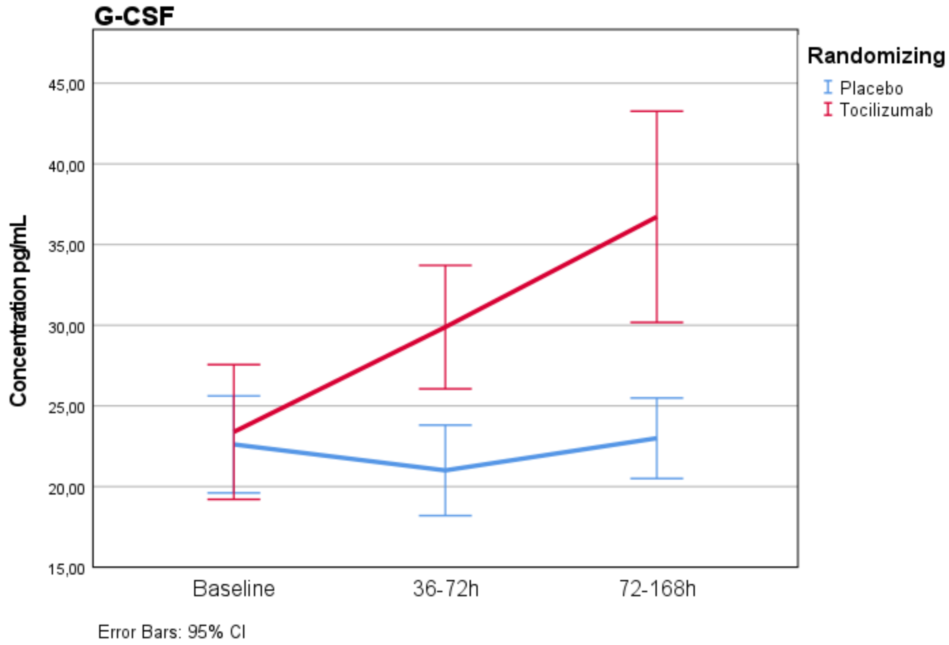

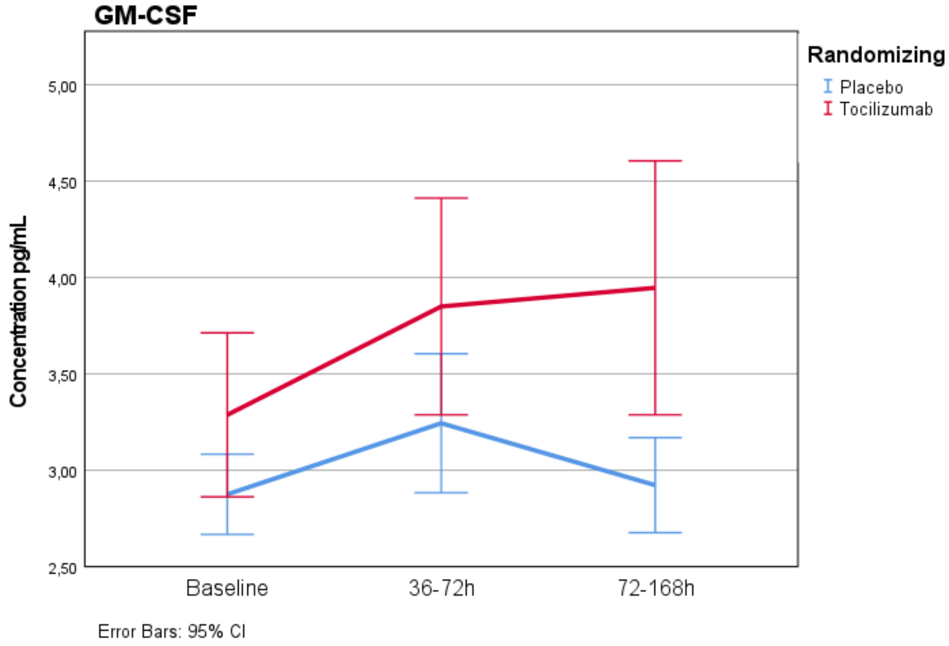
GM-CSF
Randomizing
Placebo
Tocilizumab
Concentration pg/mL
5,00
4,50
4,00
3,50
3,00
2,50
Baseline
36-72h
72-168h
Error Bars: 95% CI

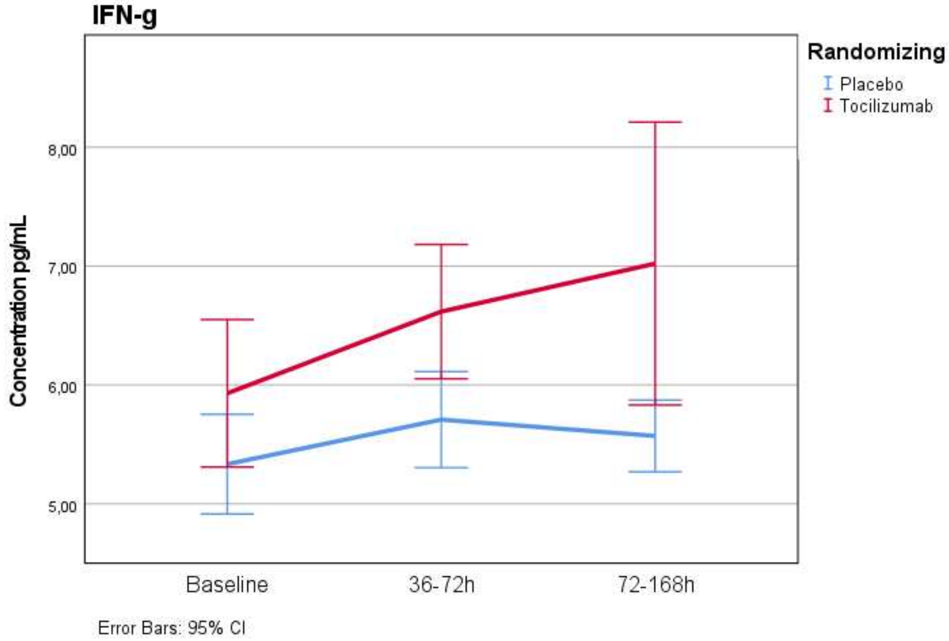
IFN-g
Randomizing
Placebo
Tocilizumab
Concentration pg/mL
8,00
7,00
6,00
5,00
Baseline
36-72h
72-168h
Error Bars: 95% CI

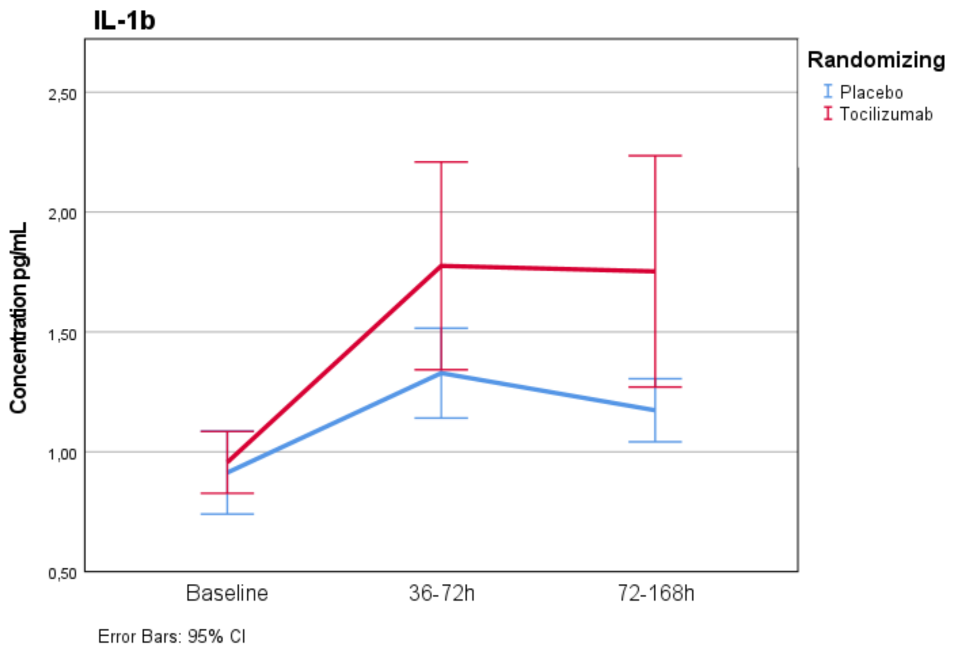
IL-1b
Randomizing
Placebo
Tocilizumab
2,50
2,00
1,50
1,00
0,50
Concentration pg/mL
Baseline
36-72h
72-168h
Error Bars: 95% CI

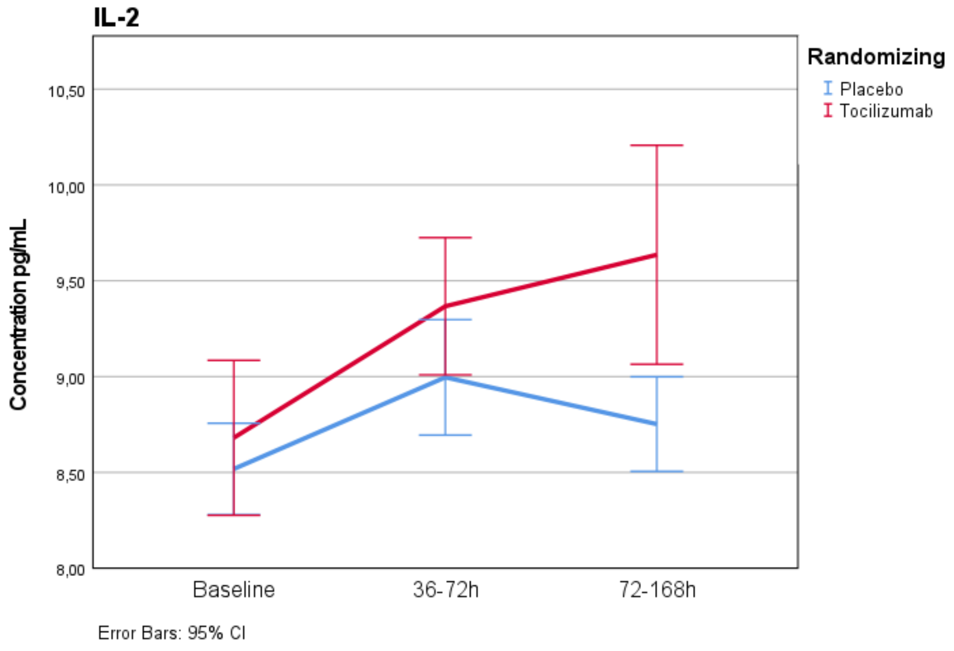
IL-2
Randomizing
Placebo
Tocilizumab
10,50
10,00
9,50
9,00
8,50
8,00
Concentration pg/mL
Baseline
36-72h
72-168h
Error Bars: 95% CI

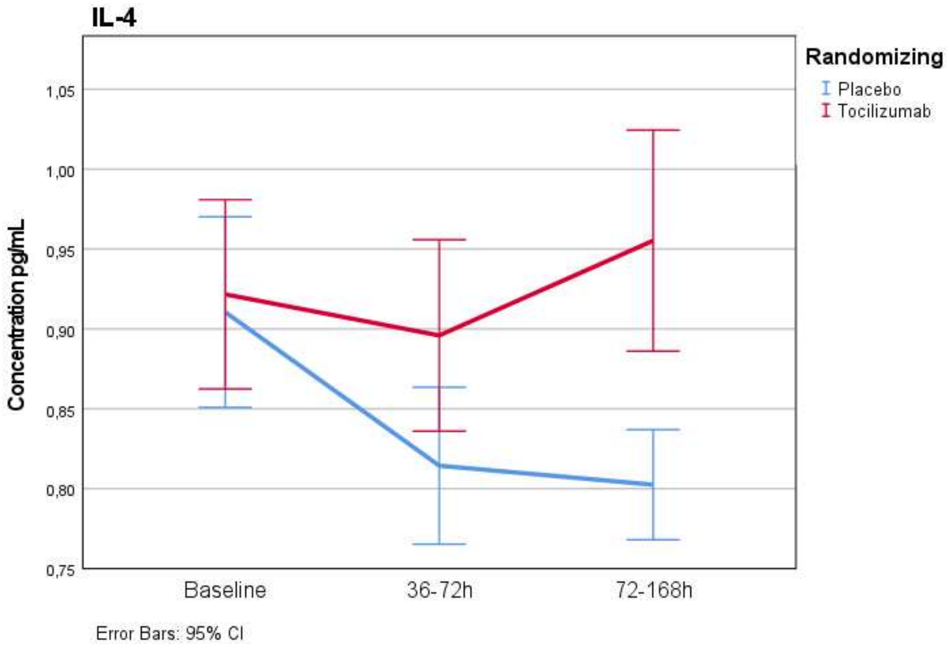
IL-4
Randomizing
Placebo
Tocilizumab
Concentration pg/mL
1,05
1,00
0,95
0,90
0,85
0,80
0,75
Baseline
36-72h
72-168h
Error Bars: 95% CI

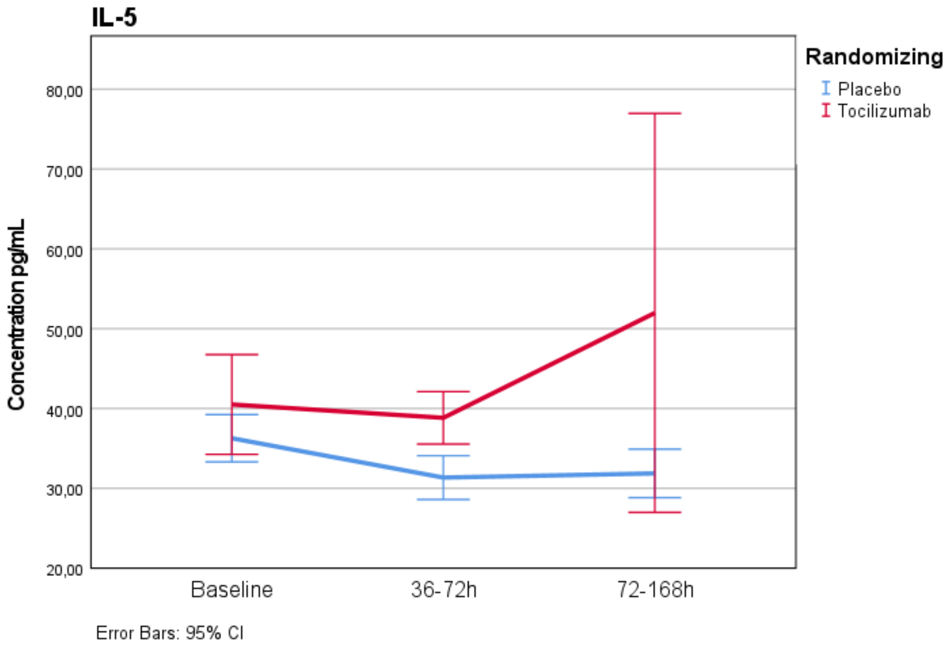
IL-5
Randomizing
Placebo
Tocilizumab
Concentration pg/mL
80,00
70,00
60,00
50,00
40,00
30,00
20,00
Baseline
36-72h
72-168h
Error Bars: 95% CI

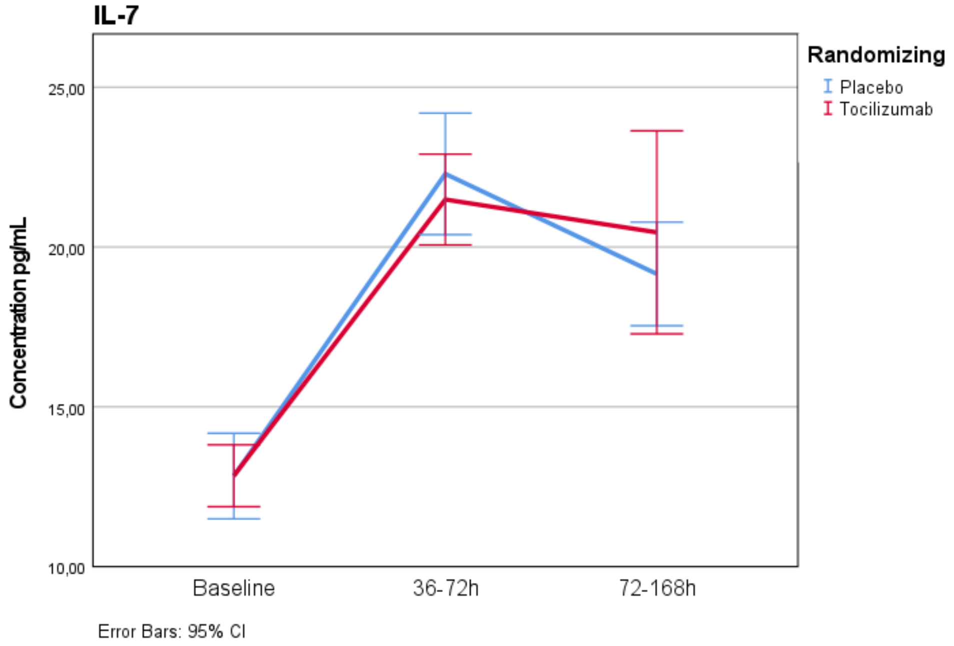
IL-7
Randomizing
Placebo
Tocilizumab
Concentration pg/mL
25,00
20,00
15,00
10,00
Baseline
36-72h
72-168h
Error Bars: 95% CI

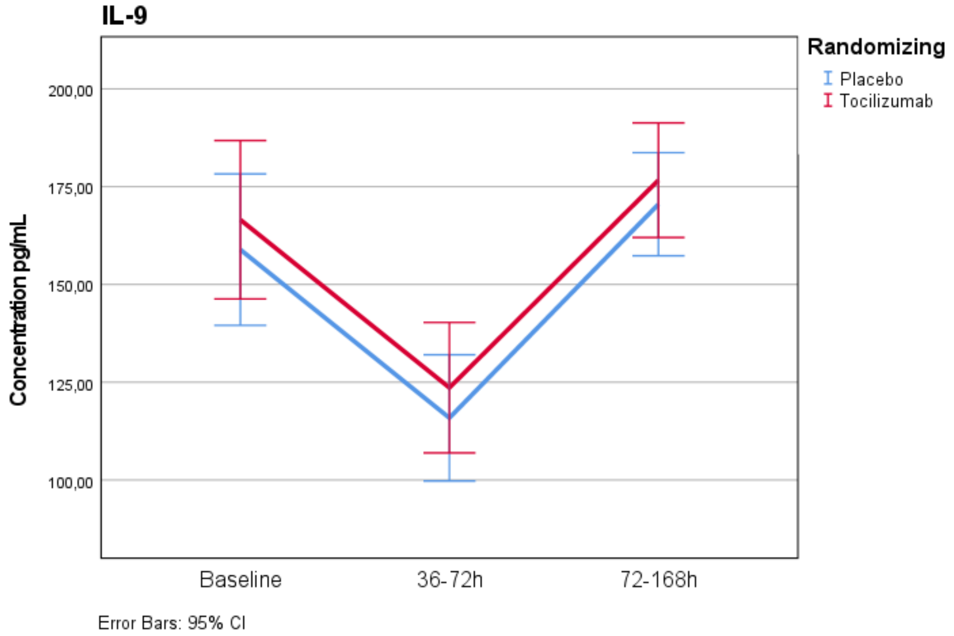
IL-9
Randomizing
Placebo
Tocilizumab
Concentration pg/mL
200,00
175,00
150,00
125,00
100,00
Baseline
36-72h
72-168h
Error Bars: 95% CI

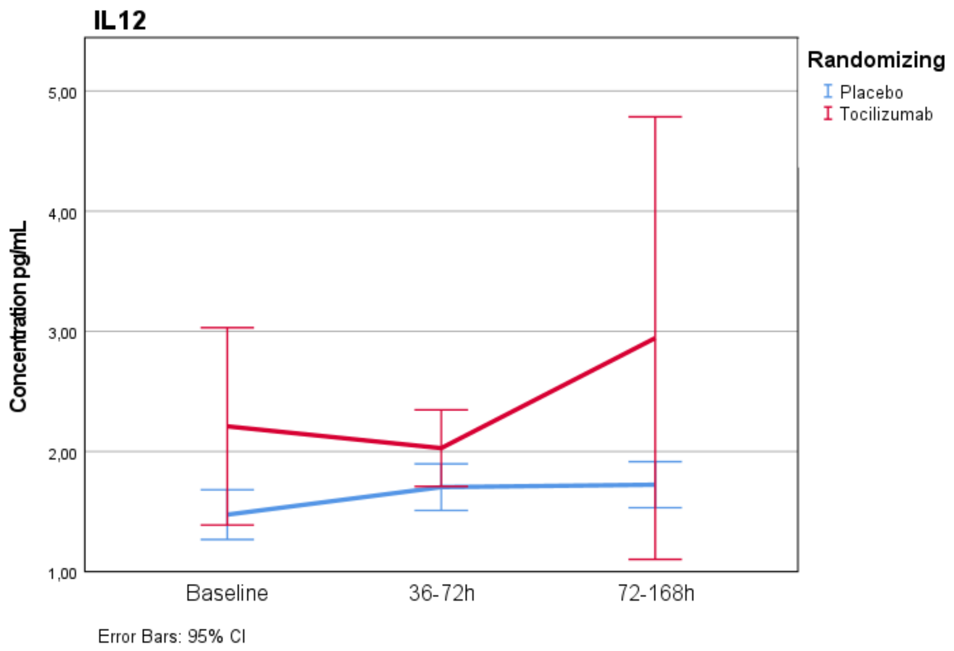
IL12
Randomizing
Placebo
Tocilizumab
Concentration pg/mL
5,00
4,00
3,00
2,00
1,00
Baseline
36-72h
72-168h
Error Bars: 95% CI

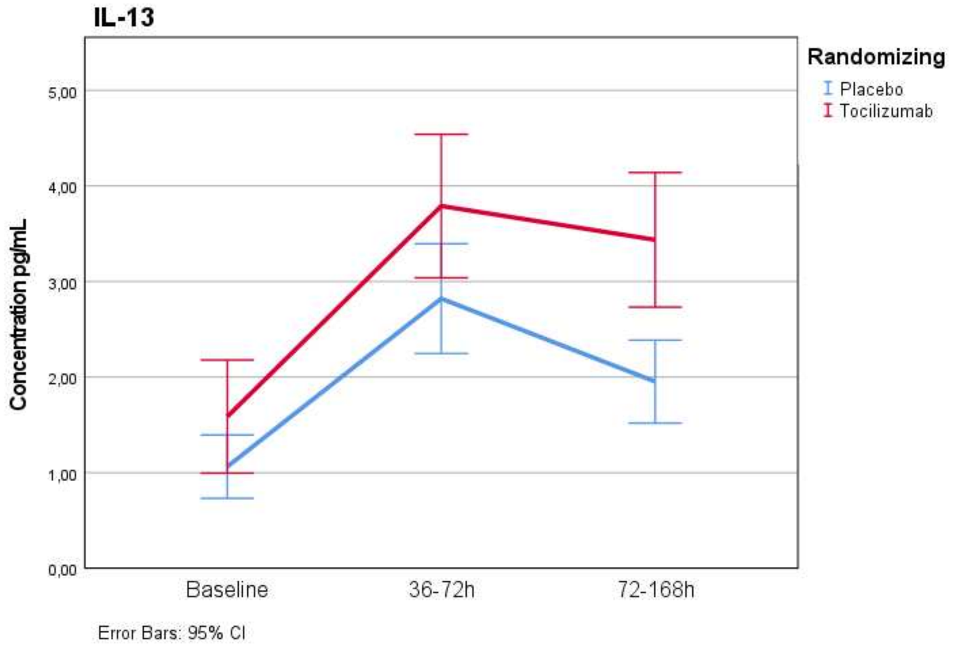
IL-13
Randomizing
Placebo
Tocilizumab
Concentration pg/mL
5,00
4,00
3,00
2,00
1,00
0,00
Baseline
36-72h
72-168h
Error Bars: 95% CI

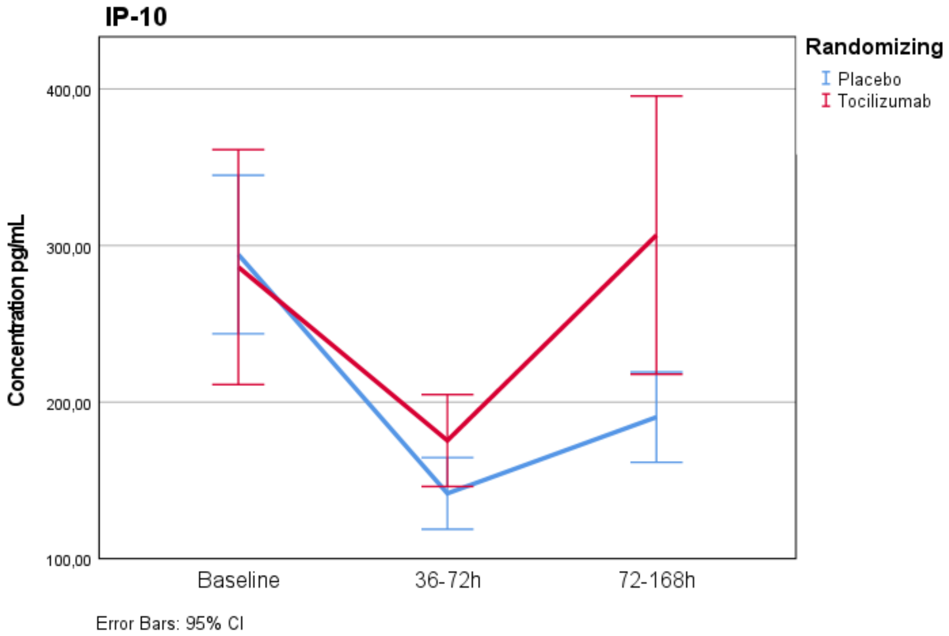
IP-10
Randomizing
Placebo
Tocilizumab
Concentration pg/mL
400,00
300,00
200,00
100,00
Baseline
36-72h
72-168h
Error Bars: 95% CI

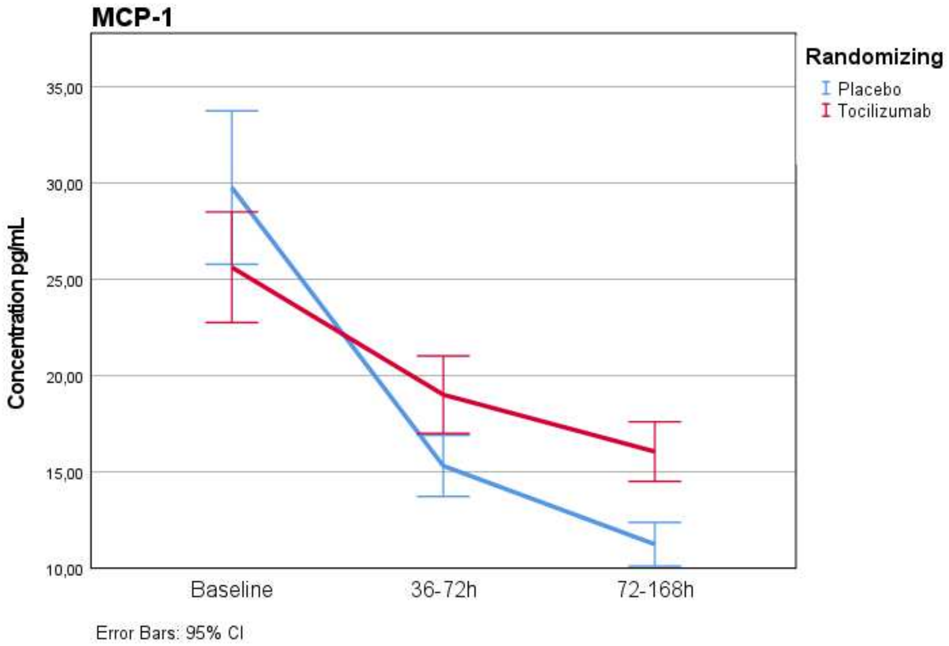
MCP-1
Randomizing
Placebo
Tocilizumab
Concentration pg/mL
35,00
30,00
25,00
20,00
15,00
10,00
Baseline
36-72h
72-168h
Error Bars: 95% CI

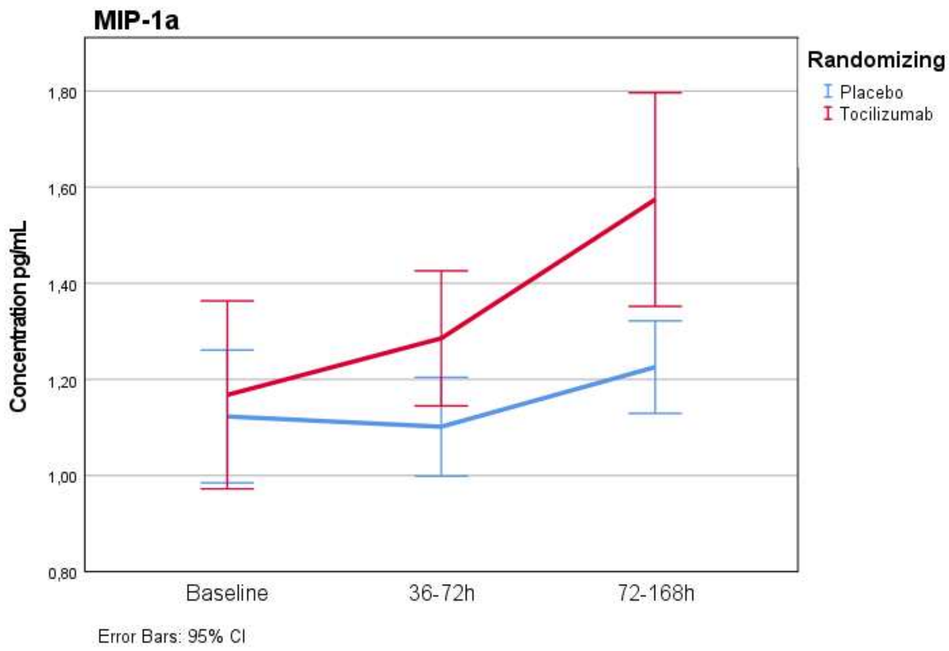
MIP-1a
Randomizing
Placebo
Tocilizumab
Concentration pg/mL
1,80
1,60
1,40
1,20
1,00
0,80
Baseline
36-72h
72-168h
Error Bars: 95% CI

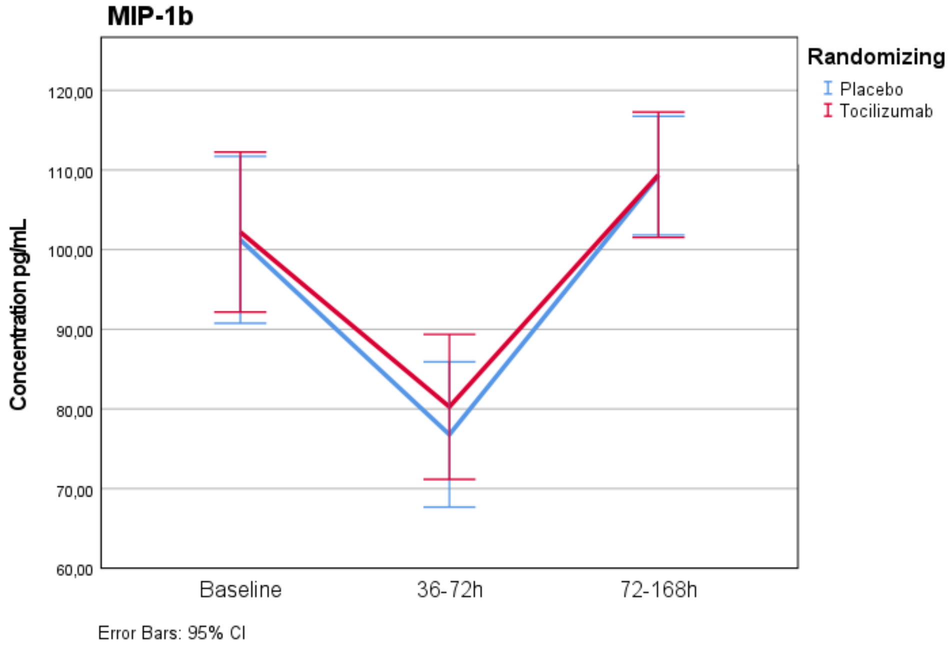
MIP-1b
Randomizing
Placebo
Tocilizumab
Concentration pg/mL
120,00
110,00
100,00
90,00
80,00
70,00
60,00
Baseline
36-72h
72-168h
Error Bars: 95% CI

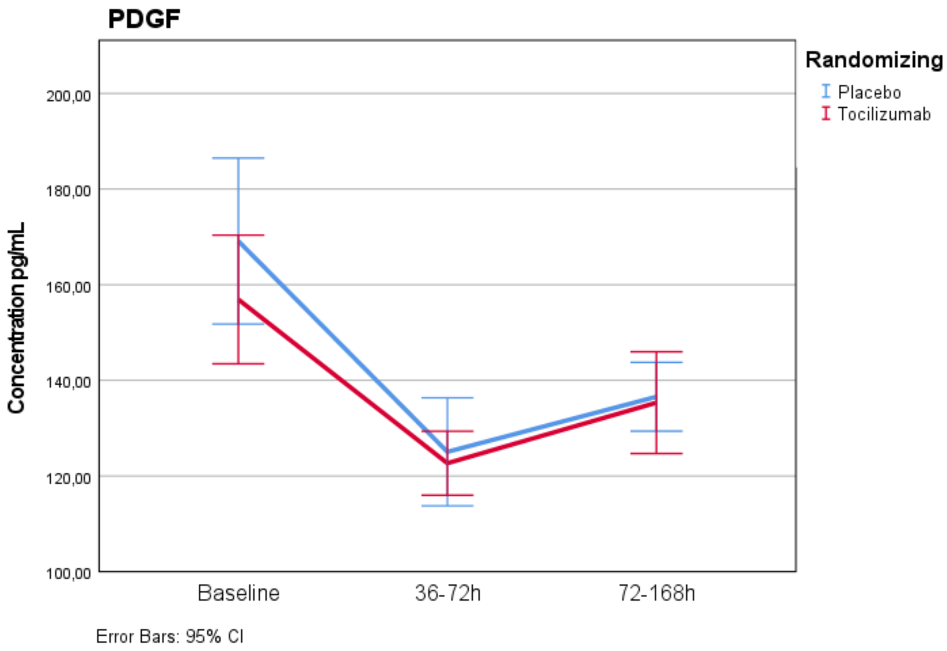

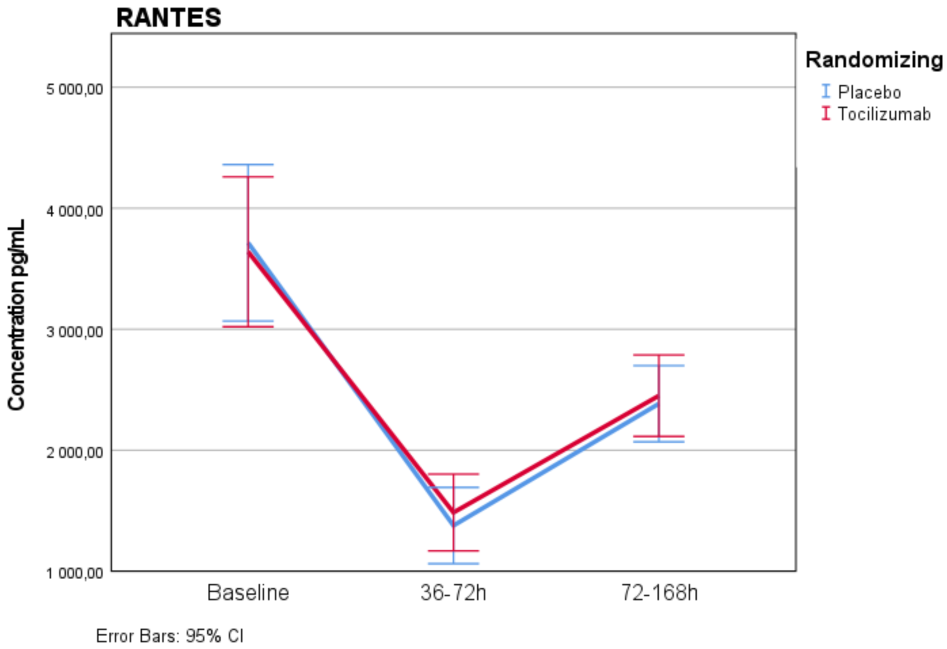

Woxholt S, et al. Open Heart 2023; 10:e002301. doi: 10.1136/openhrt-2023-002301

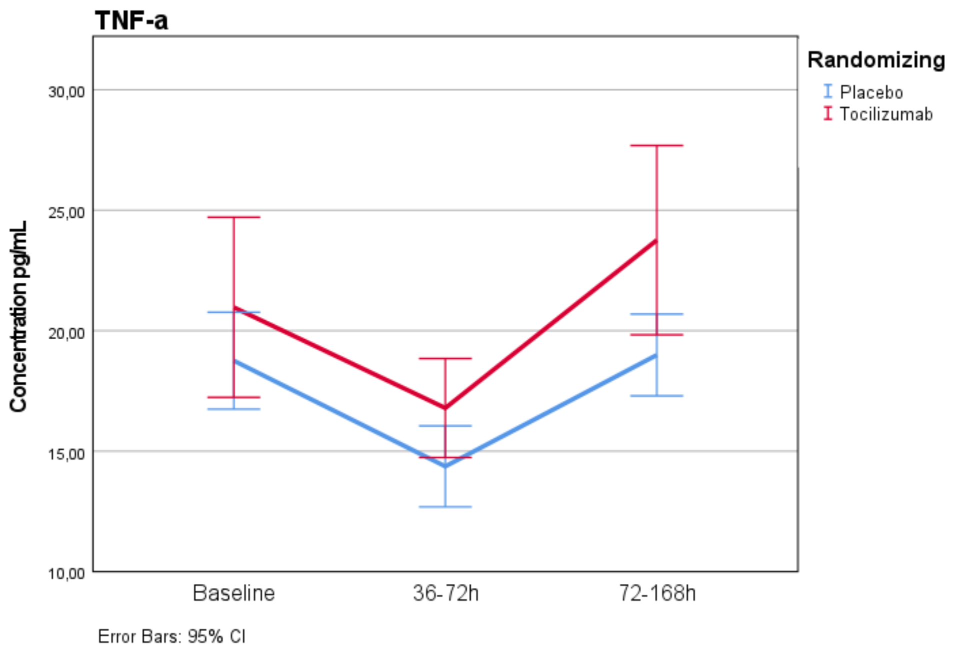

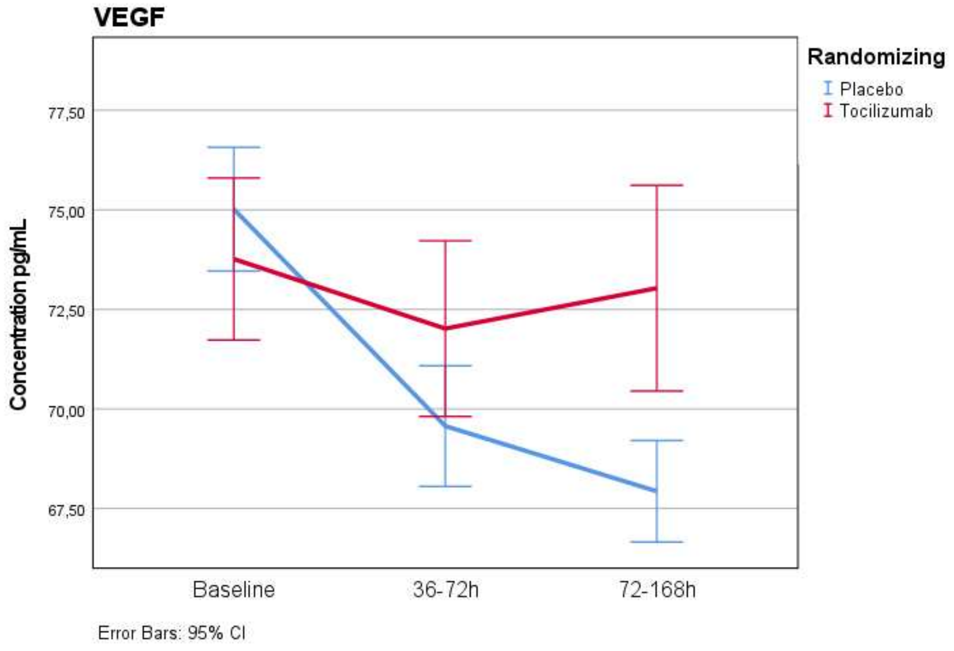

G-CSF = Granulocyte colony stimulating factor. GM-CSF = granulocyte-macrophage colony-stimulating factor. IFN-γ = Interferon-gamma. IL = Interleukin. ra = receptor antagonist. IP = interferon-γ inducible protein. MCP = macrophage chemoattractant protein. MIP = macrophage inflammatory protein. PDGF = platelet-derived growth factor. RANTES = regulated on activation, normal T cell expressed and secreted. TNF = tumor necrosis factor. VEGF = Vascular endothelial growth factor.

Woxholt S, *et al. Open Heart* 2023; 10:e002301. doi: 10.1136/openhrt-2023-002301
